# COMMD4 functions with the histone H2A-H2B dimer for the timely repair of DNA double-strand breaks

**DOI:** 10.1038/s42003-021-01998-2

**Published:** 2021-04-19

**Authors:** Amila Suraweera, Neha S. Gandhi, Sam Beard, Joshua T. Burgess, Laura V. Croft, Emma Bolderson, Ali Naqi, Nicholas W. Ashton, Mark N. Adams, Kienan I. Savage, Shu-Dong Zhang, Kenneth J. O’Byrne, Derek J. Richard

**Affiliations:** 1 Queensland University of Technology (QUT), School of Biomedical Sciences, Centre for Genomics and Personalised Health at the Translational Research Institute, Woolloongabba, QLD Australia; 2 Queensland University of Technology (QUT), School of Chemistry and Physics, Faculty of Science and Engineering, Centre for Genomics and Personalised Health, Brisbane, QLD Australia; 3 Department of Chemistry, Pennsylvania State University, University Park, PA USA; 4 The Eunice Kennedy Shriver National Institute of Child Health and Human Development, National Institutes of Health, Bethesda, MD USA; 5 The Patrick G Johnston Centre for Cancer Research, Queen’s University Belfast, Belfast, UK; 6 Northern Ireland Centre for Stratified Medicine, University of Ulster, Londonderry, UK; 7 Princess Alexandra Hospital, Woolloongabba, QLD Australia

**Keywords:** DNA damage response, Molecular biology, Ubiquitylation

## Abstract

Genomic stability is critical for normal cellular function and its deregulation is a universal hallmark of cancer. Here we outline a previously undescribed role of COMMD4 in maintaining genomic stability, by regulation of chromatin remodelling at sites of DNA double-strand breaks. At break-sites, COMMD4 binds to and protects histone H2B from monoubiquitination by RNF20/RNF40. DNA damage-induced phosphorylation of the H2A-H2B heterodimer disrupts the dimer allowing COMMD4 to preferentially bind H2A. Displacement of COMMD4 from H2B allows RNF20/40 to monoubiquitinate H2B and for remodelling of the break-site. Consistent with this critical function, COMMD4-deficient cells show excessive elongation of remodelled chromatin and failure of both non-homologous-end-joining and homologous recombination. We present peptide-mapping and mutagenesis data for the potential molecular mechanisms governing COMMD4-mediated chromatin regulation at DNA double-strand breaks.

## Abstract (web summary)

Amila Suraweera et al. use a range of biochemical and in vitro cellular assays to examine the role of the COMMD4 in DNA repair. Their results suggest that COMMD4 interacts with the histone H2A-H2B during repair of double-stranded DNA breaks, thereby maintaining genomic stability by regulating chromatin structure.

## Introduction

Our cells encounter thousands of DNA damaging events each day. If left unrepaired, this damage can lead to genomic instability or malignant transformation^1,2^. DNA double-strand breaks (DSBs) are amongst the most toxic lesions to cells, as their incorrect repair can lead to chromosomal fragmentation and cell death^2–4^. Cells repair DSBs by two major pathways: non-homologous end-joining (NHEJ) and homologous recombination (HR). NHEJ re-joins broken DNA ends without the use of extensive homology and is often regarded as being error-prone. In contrast, HR repairs DSBs with high fidelity, although as it requires a sister chromatid for a repair template, it can only be used during the S and G2 cell cycle phases^1,5,6^. Regardless of the repair pathway, the chromatin surrounding the break-site must be remodelled to allow access of DNA repair proteins^7,8^.

Chromatin remodelling plays an essential role in the functioning of all cellular processes involving DNA, including transcription, DNA replication and DNA repair and involves the concerted action of histone post-translational modifications, including, phosphorylation, methylation, acetylation and ubiquitination^9^. Such responses upon the induction of DNA DSBs include the phosphorylation and ubiquitination of H2AX, as well as the ubiquitination of H2A by RNF8 and RNF168. These chromatin modifications function to recruit DNA repair proteins to the sites of DSBs and to initiate the DNA damage response, resulting in altered transcriptional and translational regulation, as well as activation of cellular checkpoints and DSB repair^10–12^.

Copper metabolism gene MURR1 (COMM) domain proteins are a family of interacting partners of COMMD1 (MURR1). All ten COMMD family members are highly conserved in multicellular eukaryotic organisms and are characterised by a carboxyl-terminal COMM domain that functions as an interface for protein–protein interactions^13^. While predicted to function in copper metabolism, the precise functional role(s) of most family members has not been fully elucidated. COMMD1 is the most extensively characterised COMMD protein and is a pleiotropic factor, regulating many biological processes, including copper and cholesterol homoeostasis, ionic transport, protein aggregation, protein trafficking, oncogenesis, and oxidative stress^14^. Futhermore, COMMD1 was recently shown to function in the repair of DSBs and be a potential therapeutic target for the treatment of non-small cell lung cancer^15^. COMMD4, the subject of this study, has been previously shown to control NFkB activity in mammals^16^, to interact with cullins and modulate the activity of cullin-RING E3 ubiquitin ligase complexes^17^ and be a good prognostic marker and therapeutic target in non-small cell lung cancer^18^. The authors demonstrated that depletion of COMMD4 in non-small lung cancer patient cell lines impaired proliferation of these cells and resulted mitotic catastrophe and cancer cell death^18^.

Here, we report a role for COMMD4 in maintaining genomic stability. COMMD4-deficient cells are genomically unstable, hypersensitive to DNA damaging agents and are unable to efficiently repair DSBs. Our data demonstrates a specific role for COMMD4 in regulating the monoubiquitination of H2B at sites of DNA DSBs, by binding to the histone H2A-H2B dimer.

## Results

### COMMD4 is required for cell survival following exposure to DNA damaging agents

Using connectivity mapping^19^, we identified that the transcript of COMMD4 was co-regulated with that of the DNA repair protein, human single-stranded DNA-binding protein 1 (hSSB1) (*p* < 0.05). We focused on COMMD4 as it was the top-ranking protein with an unknown functional role.

**Fig. 1:**
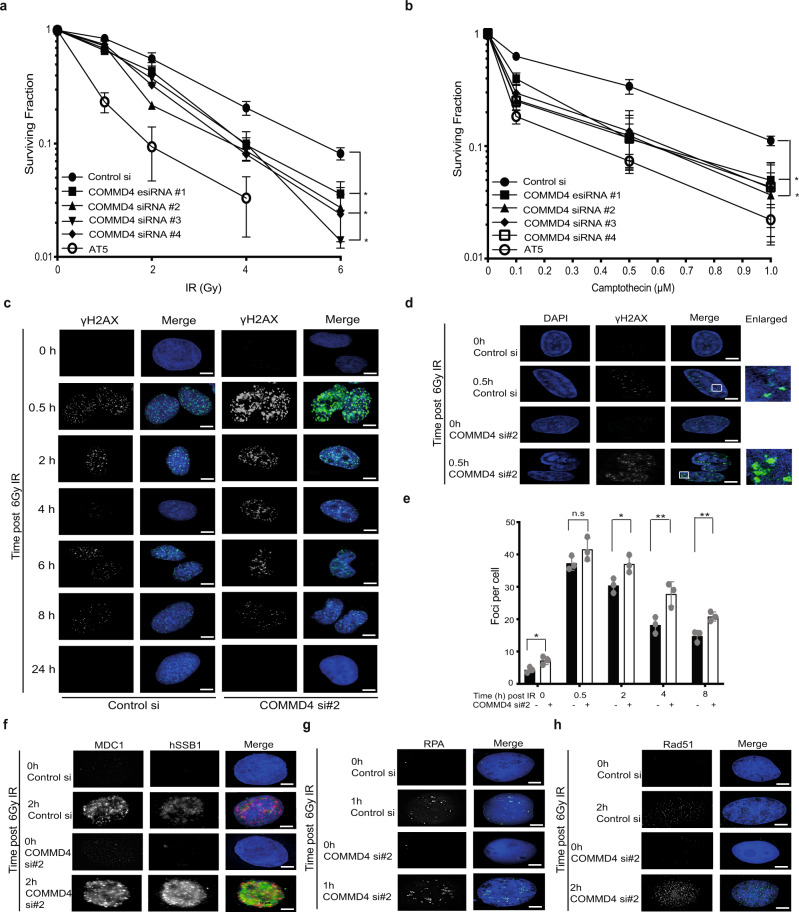
Loss of COMMD4 results in genomic instability. **a**, **b** U20S cells were transfected with COMMD4 esiRNA (#1) and/or siRNA #2, siRNA #3 and siRNA #4 to deplete COMMD4 protein levels and treated with varying doses of IR or CPT. A clonogenic assay was subsequently carried out to determine the sensitivity of COMMD4-depleted cells to these agents. AT5; cells from an A-T patient. Asterix (*) denotes *p* < 0.05. Error bars represent mean ± SD from three independent experiments. **c** Immunofluorescence on control and COMMD4-depleted U2OS cells treated with 6 Gy of IR followed by recovery at the time points shown and stained with γH2AX. **d** γH2AX foci in control and COMMD4-depleted cells using the OMX super-resolution microscope. **e** Plot of γH2AX foci numbers for **c** and **d**. **f** Immunofluorescence experiment demonstrating MDC1 and hSSB1 foci formation in control and COMMD4-depleted cells before and 2 h after irradiation. **g**, **h** Immunofluorescence experiment demonstrating RPA and Rad51 foci formation respectively in control and COMMD4-depleted cells before and after irradiation. DAPI shows the nucleus. ns not significant where *p* > 0.05, *p < 0.05 and **p < 0.005. Error bars represent mean ± SD from three independent experiments where 50 cells were quantified per condition. Scale bar denotes 5 μm.

The function of hSSB1 is critical for genome stability, functioning in the repair of DSBs by HR^20–22^. To explore if COMMD4 functions similarly, we used an esiRNA and three different siRNA sequences to deplete COMMD4 from cells (Supplementary Figs. 1a and 11a); we then treated these cells with DNA damaging agents. Cells depleted of COMMD4 exhibited increased sensitivity to DNA damaging agents (Fig. 1a, b). We used three different siRNA sequences to discount siRNA off-target-effects and rescued the sensitivity to one of the siRNA sequences (siRNA #2) by transient expression of siRNA-resistant COMMD4 (Supplementary Figs. 1b, c and 11b). Since COMMD4-FLAG partially corrected the defect in cells depleted of COMMD4, we used siRNA #2 for the remainder of our experiments.

### COMMD4 is required for the repair of DNA DSBs

Following induction of DSBs, hSSB1 rapidly localises to break-sites where it recruits the MRN complex to allow ATM activation^20–22^. To explore whether COMMD4 functions similarly, we depleted U2OS cells of COMMD4 using siRNA and used immunofluorescence to visualise the phosphorylation of histone H2AX (γH2AX), an indirect marker of DSBs^23,24^, following ionising radiation (IR) exposure. In COMMD4-depleted cells, γH2AX signals were greatly enhanced, both prior to and post-DSB induction (Fig. 1c and Supplementary Fig. 1d), with the defect partially rescued by ectopic expression of COMMD4-FLAG (Supplementary Fig. 1e–g). This was further confirmed by immunoblotting chromatin fractions of IR-treated cells (Supplementary Figs. 1h and 11c). To further explore how COMMD4-depletion contributes to γH2AX foci formation, we performed structured illumination microscopy (SIM), which resolved structures down to 120 nm. This revealed that the enhanced γH2AX signal was due to an increase in both the number and average foci size of γH2AX (Fig. 1d, e). Importantly, the average signal per unit area of each foci was unchanged, suggesting larger γH2AX represented abnormal extension from the break-site. Consistent with these findings, we also observed increased chromatin-bound hSSB1 and MDC1^25^ signals (Fig. 1f and Supplementary Fig. 2a), as well as increased RPA^26^ and Rad51^27^ foci (Fig. 1g, h and Supplementary Fig. 2b) in COMMD4-depleted cells, in-line with the presence of more DSBs and larger repair foci.

Once a DSB is induced, the cell initiates signalling events to activate cellular checkpoints and DSB repair^1^. HEK293T cells depleted of COMMD4 demonstrated enhanced p53 S15 and Chk2 T68 phosphorylation, consistent with increased activation of the checkpoint (Supplementary Figs. 2c and 12).

**Fig. 2:**
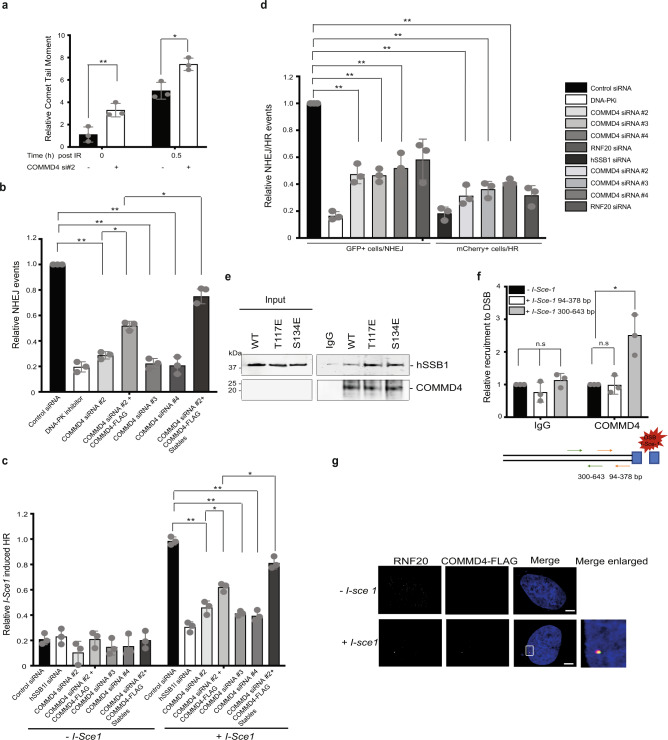
Repair of DNA double-strand breaks in COMMD4-depleted cells and interaction with hSSB1. **a** Comet assay showing the relative comet tail moment in COMMD4-deficient and control cells at 0 and 0.5 h post treatment with 6 Gy IR. **b** Plot of the relative NHEJ events in control, cells treated with DNA-PK inhibitor, COMMD4-depleted cells, COMMD4-depleted cells transiently overexpressing COMMD4-FLAG and cells stably expressing COMMD4-FLAG, 48 h post-transfection with HindIII linearised EGFP-N3 plasmid. **c** Plot of *I-Sce1* induced HR in control, hSSB1-depleted, COMMD4-deficient cells and COMMD4-deficient cells overexpressing COMMD4-FLAG transiently and stably. **d** Plot of the relative NHEJ and HR events, respectively, in control, DNA-PK inhibitor, hSSB1 siRNA and COMMD4-depleted cells using a quantitative reporter assay that measures NHEJ versus HR in the same cells through the repair of two inverted *I-Sce1* cuts. **e** A direct interaction between COMMD4 and WT hSSB1, T117E and S134E hSSB1. COMMD4 was used to pull-out the protein complexes. IgG is the negative control and COMMD4 shows the loading. **f** ChIP-qPCR assay with primers at 94–378 and 300–643 bp from the break-site in DRGFP cells transfected with COMMD4-FLAG, ± *I-Sce1*. The enrichment of COMMD4 after induction of a DSB was compared to the IgG control and - *I-Sce1* sample. Significant recruitment was only observed at 300–643 bp. The schematic of the assay and the primer pairs used is shown below. *p < 0.05, **p < 0.005, ns non-significant where *p* > 0.05. Error bars represent mean ± SD from three independent experiments. **g** Immunofluorescence showing localisation of FLAG-COMMD4 and RNF20 at a DSB induced by the *I-SceI* restriction enzyme in MCF7 DRGFP cells. Scale bar denotes 5 μm.

To confirm that COMMD4-depleted cells have greater numbers of DSBs, we used neutral COMET assays to directly observe DNA fragmentation^28^. This showed that cells depleted of COMMD4 had greater numbers of DSBs prior to and following IR-exposure, compared to controls (Fig. 2a and Supplementary Fig. 3a). We next analysed the activity of the two DSB repair pathways, NHEJ and HR, using previously described in-cell assays^20,29–31^. We initially measured NHEJ using a GFP-based assay where the pEGFP-N1 vector was used as the reporter^29,30^ (Fig. 2b), subsequently measured HR in the MCF7 cell line, stably integrated with a GFP-based HR reported^26^ (Fig. 2c) and both NHEJ and HR together in U20S cell stably integrated with GFP and mCherry-based reporters^31^ (Fig. 2d). These assays indicated that both NHEJ and HR were functionally impaired in the absence of COMMD4 (Fig. 2b–d), with a partial rescue by COMMD4-FLAG (Fig. 2b, c) and a near complete rescue in stable HEK293 cells expressing COMMD4-FLAG. This demonstrated that COMMD4 function was required for a common processing step required in both NHEJ and HR.

### Subcellular localisation of COMMD4 and its recruitment to the sites of damage

To determine whether COMMD4 functions directly in the repair process, we next examined if COMMD4 localises to break-sites. We detected endogenous COMMD4 in the nucleus by immunoblotting of subcellular fractions (Supplementary Figs. 3b and 13). Immunofluorescence in U2OS cells ectopically expressing COMMD4-FLAG revealed nuclear and cytoplasmic staining (Supplementary Fig. 3c), supporting our subcellular fractionations. Interestingly, γH2AX did not directly co-localise with COMMD4-FLAG; it was however found immediately adjacent to over 50% of γH2AX foci. This was further corroborated by immunofluorescence of endogenous COMMD4 in U2OS cells (Supplementary Fig. 3d, f). COMMD4 foci were also adjacent to, but not overlapping with 53BP1 foci^32^ (Supplementary Fig. 3e, f). The adjacent localisation between COMMD4 and 53BP1 foci further demonstrates that COMMD4 may be required for correct remodelling of chromatin which precedes both γH2AX and 53BP1 foci formation.

We next investigated how COMMD4 is recruited to break-sites and if its recruitment is mediated by hSSB1. Co-immunoprecipitation assays revealed that COMMD4 and hSSB1 form a complex in cells (Supplementary Figs. 4a and 14a) and in vitro pull-down assays using purified COMMD4 and hSSB1 showed a direct interaction (Fig. 2e and Supplementary Fig. 8). We included T117E and S134E phosphomimetic mutants of hSSB1, which represent the damage-induced phosphorylation of hSSB1 by ATM^20^ and DNA-PK^33^, respectively. These data suggest that while hSSB1 and COMMD4 interact regardless of hSSB1 phosphorylation, their interaction is enhanced by phosphorylation of hSSB1 at T117/S134. We next used siRNA to deplete hSSB1 from cells and assessed COMMD4 foci formation by immunofluorescence (Supplementary Fig. 4b). The reduced formation of IR-induced COMMD4 foci in the absence of hSSB1 indeed support a requirement for hSSB1 in the recruitment of COMMD4 to sites of DSBs, likely involving a direct interaction between COMMD4 and phosphorylated hSSB1. Furthermore, the ATM inhibitor KU-55933 demonstrated reduced formation of IR-induced COMMD4 foci (Supplementary Fig. 4c).

We next determined whether COMMD4 localised to a site-specific DBS using a chromatin immunoprecipitation (ChIP) assay with DRGFP cells previously described (Fig. 2c) and overexpressed COMMD4-FLAG transiently. *I-Sce1* was used to introduce a site-specific DSB and FLAG antibody was employed to immunoprecipitate COMMD4. The enrichment of COMMD4 after the induction of a DSB was compared to the IgG control and the - *I-Sce1* sample, using two primer pairs; one immediately adjacent to the break-site and the other at 300 bp or greater away from the break-site^20,34^ (Fig. 2f). The ChIP assay demonstrated that COMMD4 specially localised to a site within 300–643 bp away from the *I-Sce1*-induced DSB. This data further demonstrate that COMMD4 precipitates and is enriched in the DNA that is at least 300 bp away from the break-site. hSSB1 was previously seen at 94–378 bp from the *I-Sce1* break-site^20^. We also observed the co-localisation of COMMD4 and RNF20 at a site-specific DSB by the *I-SceI* restriction enzyme in the DRGFP cells (Fig. 2g).

### COMMD4 associates with the histone, H2B and the E3 ubiquitin ligase complex, RNF20/40

Following DSB induction, the chromatin surrounding the break-site must be remodelled. Part of this remodelling involves the monoubiquitination of H2B by the ubiquitin ligase RNF20/40 complex in an ATM-dependent manner; a process required for the efficient repair of DSB by HR and NHEJ^35–37^. This monoubiquitination of H2B has been shown to disrupt chromatin compaction and inter-fibre interactions, leading to an open and accessible chromatin fibre^38^.

**Fig. 3:**
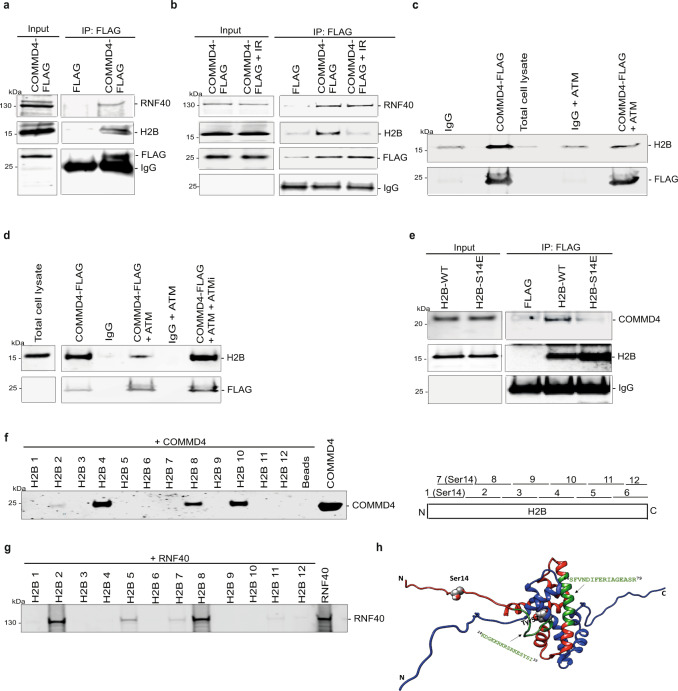
COMMD4 interacts with H2B and RNF40. **a** Empty vector (FLAG) and COMMD4-FLAG were immunoprecipitated from HeLa cells and the co-eluting proteins were immunoblotted with antibodies shown. **b** Immunoprecipitation as **a**, but this time cells were treated with 6 Gy IR. **c** A direct interaction for COMMD4 and H2B ± recombinant ATM. **d** A direct interaction between COMMD4 and H2B ± recombinant ATM and the ATM inhibitor (ATMi) KU-55933. **e** FLAG and FLAG-tagged H2B WT, and S14E mutant were immunoprecipitated from HeLa cells and the co-eluting proteins were immunoblotted with antibodies against COMMD4 and H2B. IgG heavy chain shows the loading. **f** Direct interaction between COMMD4 and H2B demonstrating the specific binding region within H2B. No binding was seen in the negative control with only the beads and recombinant COMMD4 (beads), while expression of His-tagged COMMD4 (COMMD4, right most lane) is seen. Schematic on the right shows peptide regions within H2B and Supplementary Table 1 shows the peptide sequences. **g** Direct interaction between RNF40 and H2B demonstrating the specific binding regions within H2B. Expression of recombinant RNF40 is seen in the control lane. **h** A three dimensional structure (PDB code 2RVQ) of the heterodimer H2A-H2B wherein H2A and H2B are shown in blue and red ribbon representation, respectively. Tails of histones are disordered and in extended conformation. The phosphorylation sites are shown as spheres.

To determine if COMMD4 is involved in chromatin remodelling at break-sites, we investigated whether COMMD4 associated with the histone H2B and the E3 ubiquitin ligase complex RNF20/40. We immunoprecipitated FLAG-tagged COMMD4 from HEK293 cells stably expressing COMMD4-FLAG, with M2 magnetic agarose beads and immunoblotted for H2B, RNF40 and COMMD4 (Fig. 3a and Supplementary Fig. 9a). These experiments demonstrated that COMMD4 associated with both H2B and RNF40. However, following irradiation, while the interaction between COMMD4 and RNF40 did not change, COMMD4 and H2B dissociated (Fig. 3b and Supplementary Fig. 9b). An in vitro binding assay, using purified proteins, additionally demonstrated that COMMD4 interacted directly with RNF20 and RNF40 (Supplementary Figs. 4d–f and 14b, c).

### Characterising the interaction between COMMD4, H2B and RNF20/40

Following the induction of a DSB, H2B is phosphorylated at Ser14 and occurs downstream of ATM signalling^39^. The dissociation of COMMD4 from phosphorylated H2B suggests that COMMD4 may protect H2B from monoubiquitination by RNF20/40. To investigate whether COMMD4 binds directly to H2B and if ATM regulates this binding, we incubated purified COMMD4-FLAG with recombinant H2B and utilised M2 magnetic agarose to pull-down COMMD4 bound to H2B. As H2B has been previously shown to be phosphorylated following DSB formation^39^, we performed this assay in the presence of recombinant ATM. These data showed that COMMD4 bound directly to unmodified H2B and that this interaction was disrupted by the presence of active ATM (Fig. 3c and Supplementary Figs. 4g and 9c). The addition of the ATM kinase inhibitor, KU-55933, restored the interaction between COMMD4 and H2B (Fig. 3d and Supplementary Figs. 4h and 9d), highlighting that it is the phosphorylation of H2B at Ser14 that disrupts their interaction. We next purified an S14E phosphomimetic mutant of H2B and repeated the pull-down assay with COMMD4 (Fig. 3e and Supplementary Fig. 9e). As expected, we were unable to detect an association between COMMD4 and S14E H2B.

We next used a peptide-mapping strategy to determine the exact site of interaction between COMMD4 and H2B. H2B was reduced to a series of 20 amino acid partially overlapping peptides containing a C-terminal Biotin tag (Supplementary Table 1) and we performed pull-down assays between COMMD4 and each peptide (Fig. 3f and Supplementary Fig. 9f). These assays demonstrated that COMMD4 bound specifically to the sequences 2, 4, 8 and 10 on H2B, as no binding was seen in the negative control with only the beads plus recombinant COMMD4.

We next used peptide-mapping to determine the site of interaction between RNF20/40 and H2B. RNF40 (Fig. 3g and Supplementary Fig. 9g) specifically bound to the sequences 2, 5, 7, 8, 11 and 12 of H2B, while RNF20 (Supplementary Figs. 4i and 14d) bound to the sequences 2, 5, 7 and 8. This demonstrated that RNF20/40 bound preferentially to KDGKKRKRSRKESYSI of H2B (Fig. 3h), as the H2A-H2B dimer structure (NMR) (PDB code: 2RVQ) provided us with two potential COMMD4 binding regions, amino acids 25–44 and 61–80. As RNF20/40 bound weakly to peptides 1 and 7 of H2B (which includes the Ser14 phosphorylation site), we next sought to determine whether the phosphorylation of H2B on Ser14 would increase RNF40 binding to the H2B peptides 1/7 (Supplementary Figs. 4j and 14e). In the presence of ATM, we observed increased binding between RNF40 and H2B peptide 7, indicating that phosphorylation of H2B at Ser14, while displacing COMMD4, recruits RNF20/40. To confirm this was due to the S14 phosphorylation of H2B, we incubated recombinant RNF40 with recombinant H2B or the S14E phosphomimic mutant and saw increased binding of RNF40 to the S14E mutant, confirming the importance of this phosphorylation event (Supplementary Figs. 4k and 14f). We additionally used FLAG-tagged COMMD4 mutants (Supplementary Table 2) to further confirm the importance of the N-terminal domain of COMMD4 for its function. These mutants of COMMD4 showed reduced binding with H2B (Supplementary Figs. 4l and 14g) and reduced NHEJ and HR repair compared to the overexpression of wild-type COMMD4 (Supplementary Fig. 4m).

### COMMD4 regulates the monoubiquitination of H2B

**Fig. 4:**
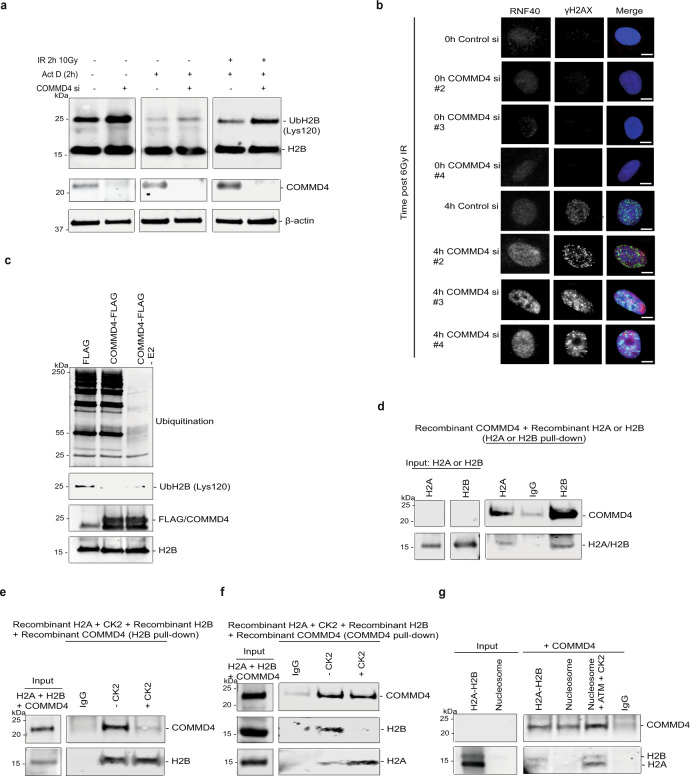
COMMD4 binds to histone H2A-H2B and regulates the monoubiquitination of H2B. **a** Immunoblot showing H2B monoubiquitination in control and COMMD4-depleted cells ±5 μg/ml of Actinomycin D and ±irradiation. **b** Immunofluorescence showing RNF40 protein levels with 6 Gy IR at 0 and 4 h post-IR treatment in control and COMMD4-depleted cells. Scale bar denotes 5 μm. Fifty cells were quantified per condition. **c** In vitro ubiquitination assay of H2B ± recombinant COMMD4 or with COMMD4 and without the E2 enzyme. Monoubiquitination and polyubiquitination of H2B is shown. **d** Direct interaction between COMMD4 and H2A/H2B, demonstrating that COMMD4 preferentially binds H2B. **e** In vitro direct interaction between H2A, H2B and COMMD4 ± recombinant CK2. H2B was used pull-out the complexes and shows loading. **f** Same as **e**, however, COMMD4 was used to pull-out the complexes. **g** Direct interaction between H2A, H2B and COMMD4, in the absence and presence of purified nucleosomes, ±recombinant ATM and CK2. COMMD4 was used pull-out the complexes and shows the loading.

In undamaged cells, the RNF20/40 complex is involved in H2B monoubiquitination during transcription elongation^40^, making it difficult to observe DSB-induced monoubiquitination of H2B. To overcome this, we treated cells with the transcription inhibitor, Actinomycin D, prior to IR which removes the transcription-associated monoubiquitinated H2B signal, allowing the DSB signal to be observed^36^. In the presence of Actinomycin D, COMMD4-depleted U2OS cells showed an increase in H2B monoubiquitination compared to control siRNA-depleted cells (Fig. 4a and Supplementary Fig. 10a). The monoubiquitination levels after the addition of Actinomycin D was much reduced, compared to cells not treated with Actinomycin D, as previously shown^36^. However, COMMD4-depleted cells treated with both Actinomycin D and IR showed a marked increase in H2B monoubiquitination, relative to the control siRNA cells treated with both Actinomycin D and IR, suggesting that in the absence of COMMD4, H2B is over-monoubiquitinated at DSB sites, a process that could prevent correct repair of the break (Fig. 4a and Supplementary Fig. 5a–c). Supporting this, immunofluorescence of COMMD4-depleted cells demonstrated a pronounced increase in the levels of RNF40 located at DSB sites, compared to controls, suggesting deregulation of remodelling at the break-site in COMMD4’s absence (Fig. 4b and Supplementary Fig. 5d).

These data highlight an important role for COMMD4 in the repair of DSBs in both HR and NHEJ, where COMMD4 functions to limit the activity of remodelling proteins at the DSB site. If COMMD4 were regulating RNF20/40 activity at sites, then, transient overexpression of COMMD4 could inhibit the monoubiquitination of H2B by RNF20/40 and thus inhibit both NHEJ and HR. This was indeed the case, with the ectopic expression of COMMD4-FLAG impairing both NHEJ and HR activity, presumably by preventing chromatin remodelling at DSB sites and thus explaining why complete rescue of NHEJ and HR activity in COMMD4-depleted cells was not possible (Supplementary Fig. 6). However, an siRNA-resistant stable cell line (HEK293-COMMD4-FLAG) that expressed near endogenous levels of COMMD4 was able to completely rescue COMMD4-depletion (Fig. 2b, c).

To further explore if COMMD4 was able to interact with the N-terminus of H2B and at the same time, limit the ubiquitination of H2B at the C-terminus, we performed an in vitro ubiquitination assay in the absence and presence of purified COMMD4-FLAG. COMMD-FLAG was incubated with recombinant H2B and purified recombinant components of the RNF20/40 ubiquitination complex (E1, E2, RNF20/40 (E3) and ubiquitin). We observed reduced H2B monoubiquitination in the reactions containing recombinant COMMD4, compared with the reaction without (Fig. 4c and Supplementary Fig. 10b), demonstrating that COMMD4 was capable of limiting the ubiquitination of H2B by RNF20/40 at its C-terminus in a purified protein system.

### COMMD4 binds to the histone H2A-H2B dimer

The phosphorylation of H2A by the CK2 kinase at tyrosine 57 is required for the regulation of transcription elongation^41^ and additionally affects H2B monoubiquitination and trimethylation of lysine in H3^41,42^. Phosphorylation of H2A by CK2 on this helix results in a loss of binding affinity between H2A and H2B due to steric hindrance by the phosphorylation site^43^. To confirm whether COMMD4 bound only to H2B or to both H2A and H2B, we performed an in vitro binding assay between COMMD4 and H2A or H2B, which demonstrated that COMMD4 bound to both H2A and H2B, but preferentially bound to H2B (Fig. 4d and Supplementary Fig. 10c). To next elucidate whether COMMD4 preferentially bound to H2B as a heterodimer with H2A, we carried an in vitro binding assay with recombinant H2A, H2B and COMMD4, in the absence and presence of the CK2 kinase. In the presence of CK2, we saw reduced binding between COMMD4 and H2B, demonstrating that while COMMD4 binds to H2B in the absence of H2A, it has a preference for the H2B-H2A heterodimer (Fig. 4e, f and Supplementary Fig. 10d, e). Additionally, in the presence of CK2, we saw COMMD4 now preferentially binding to H2A (Fig. 4f and Supplementary Fig. 10e). To determine whether the interaction between COMMD4 and H2A-H2B are maintained in nucleosomes, we performed in vitro binding assays between COMMD4 and either the H2A-H2B dimer, non-remodelled human native nucleosomes or nucleosomes that have been remodelled after phosphorylation by the ATM and CK2 kinases (Fig. 4g and Supplementary Fig. 10f). Our data with nucleosomes confirmed our experiments with purified recombinant H2A/H2B, demonstrating that COMMD4 does not bind to non-remodelled nucleosomes and requires the action of both ATM and CK2 kinases, after the induction of DNA damage, to gain access to the internalised H2A-H2B binding site.

Previous data^44,45^ have shown that following chromatin remodelling, transient conformations of histones may dissociate the H2A-H2B dimer from the nucleosome, thus corroborating the binding of COMMD4 to H2A-H2B, even though the histone binding site may be internally buried within the nucleosome. Our data support a role for COMMD4 in preventing RNF20/40 access to H2B until nucleosome remodelling has been initiated. This remodelling and post-translational modifications results in RNF20/40 gaining access to H2B allowing further nucleosome remodelling to occur. Interestingly, after H2A and H2B phosphorylation, COMMD4 does not leave the remodelling site, instead binding to H2A (Supplementary Fig. 7).

## Discussion

The DNA repair pathways have evolved in human cells as a means to maintain the genetic stability of a cell, preventing mutations that can result in diseases such as cancer^1,2^. Here we report a previously undescribed role for COMMD4 in maintaining genomic stability through the regulation of chromatin structure at sites of DSBs. We found that cells depleted of COMMD4 were sensitive to several DNA damaging agents that cause DSBs and are defective in their repair of DSBs. To explore this further, we utilised several assays to measure the repair of DSBs by NHEJ and HR in control and COMMD4-depleted cells, which indicated that both NHEJ and HR are functionally impaired in the absence of COMMD4. Furthermore, a COMMD4 ChIP assay demonstrated that COMMD4 localised adjacent to a DSB site, but not immediately proximal to the site.

The remodelling of chromatin at DSBs is a common feature of HR and NHEJ repair and allows signalling and repair proteins to access these sites^8^. Co-immunoprecipitation of COMMD4 and in vitro direct binding assays revealed an interaction with the histones H2A/H2B and the chromatin modifier RNF20/40, suggesting COMMD4 may also be involved in regulating chromatin structure at DSB sites. Interestingly, while we found that COMMD4 interacts directly with H2B, our data demonstrate that this interaction is disrupted by the phosphorylation of H2B at Ser14 in the presence of recombinant ATM. ATM-mediated phosphorylation of RNF20/40 has previously been shown to be required for regulating the monoubiquitination of H2B, which leads to H2B dissociation from the chromatin and ‘opening’ of the break-site for repair^35,36,38,40,46^. We and others^35,36^ have observed increased H2B monoubiquitination following IR-induced DSB formation. This is contrary to a recent publication^47^, where loss of H2B monoubiquination was observed after DSBs induced by AsiSI.

Our data suggest that COMMD4 protects H2B from RNF20/40 until its phosphorylation at serine 14. Once COMMD4 is displaced, RNF20/40 can then bind to H2B and ubiquitinate lysine 120, which in turn stimulates the release of H2B from the chromatin.

We used in vitro pull-down assays to map the interaction between H2B and COMMD4, which showed that COMMD4 bound to both H2B and the H2A-H2B dimer. However, following the induction of DNA damage, as H2B is phosphorylated, COMMD4 is displaced from H2B, allowing RNF20/40 access to monoubiquitinate H2B. Indeed, our data support that phosphorylation of H2B and H2A is the molecular switch that causes a decrease in COMMD4’s affinity for H2B, allowing COMMD4 to preferentially bind to H2A and consequently enable RNF20/40 to shift over to the Ser14 site of H2B.

Our data were further corroborated with experiments with native nucleosomes. Here we demonstrated that COMMD4 cannot bind to unperturbed chromatin as the binding site is not available. Upon the induction of DNA damage, the ATM and CK2 kinases remodel the nucleosome, enabling the binding of COMMD4.

Taken together, our findings demonstrate that COMMD4 is a critical component of the genome stability pathway, functioning to limit the extent of epigenetic modification of the chromatin around the break-site. Loss of COMMD4 results in a loss of RNF20/40 regulation and uncontrolled remodelling. The regulation of COMMD4 and RNF20/40 activity is controlled so that remodelling can be finely tuned. Since RNF20/40 are in a constitutive complex with COMMD4, this provides a mechanism whereby activation and suppression of H2B ubiquitination can be finely and rapidly controlled. While further structural insight via cryo-EM or crystallography will be needed to finely map the details of the molecular mechanism, it is likely that binding of COMMD4 to H2A places RNF20/40 proximal to H2B allowing it to effectively ubiquitinate H2B.

COMMD4 levels within the cell must be tightly controlled as both depletion and overexpression of COMMD4 can result in defects in both the NHEJ and HR. We anticipate our data will shed further light on how cells repair DSBs and in particular, how cells control repair of these breaks. We speculate that COMMD4 may become a therapeutic target for the treatment of cancer.

## Methods

### Cell lines and cell treatments

U20S, HeLa, HEK293 and HEK293T cells (ATCC) were cultured in Roswell Park Memorial Institute 1640 medium (Life Technologies). A-T patient cells (AT5)^29^ were cultured in Dulbecco’s Modified Eagle Medium (DMEM) (Life Technologies). MCF7 cells stably expressing a green fluorescent protein reporter gene (pDRGFP) were cultured in DMEM media containing 10 μg/ml insulin (Life Technologies) and U20S cells stably expressing the DNA repair reporter were cultured in DMEM media. All media contained 10% fetal bovine serum, and all cells were maintained in a humidified incubator at 37 °C/5% CO_2_. Camptothecin and Actinomycin D were purchased from Sigma-Aldrich. The ATM inhibitor (KU-55933) and DNA-PK inhibitor (KU-57788) were purchased from Selleckchem. Irradiations were carried out using a ^137^Cs source (Gammacell 40 Exactor [MDS Nordion]; dose rate 1.1 Gy/min).

### Antibodies

Primary antibodies: anti-COMMD4 (Abcam, ab115169 and Bioss, bs-8037R), anti-Histone H3 (Sigma-Aldrich, H0164), anti-nucleolin (Cell Signalling, 12247S), anti-γH2AX (Abcam, ab26350), anti-FLAG (Sigma, F1804), anti-MDC1 (Sigma, PLA0016), anti-p53 Serine 15 (Cell Signalling, 9284), anti-p53 clone D0-7 (Sigma-Aldrich, p8999), anti-Chk2 Threonine 68 (Cell Signalling, 2661), anti-Chk2 (Cell Signalling, 2662), anti-ATM Serine 1981 (Cell Signalling, 13050), anti-ATM (Cell Signalling, 2873), anti-β-actin (BD Biosciences, 612656), anti-H2B (Abcam, ab1790), anti-ubiquityl-histone H2B (Lys120) (Cell Signalling, 5546), anti-RNF20 (Abcam, ab32629 and Merck, MABE948), anti-RNF40 (Abcam, ab126959), anti-H2A (Abcam, 88770), anti-53BP1 (Merck-Millipore, MAB3802), anti-ubiquitin (Cell Signalling, 3936) and anti-hSSB1^20–22^. For co-immunoprecipitations and in vitro assays, the following IgG controls were used; normal rabbit IgG (Cell Signalling, 2729), normal mouse IgG (Sigma-Aldrich, N103) and normal sheep IgG (Sigma-Aldrich, 12-515).

Secondary antibodies for immunoblotting: IRDye^®^ 680CW Donkey anti-rabbit, IRDye^®^ 800CW Donkey anti-mouse and IRDye^®^ 800CW Donkey anti-goat from LI-COR, Inc.

Secondary antibodies for immunofluorescence: Alexa Fluor^®^ 488 donkey anti-mouse (A21202), Alexa Fluor^®^ 488 donkey anti-rabbit (A21206), Alexa Fluor^®^ 594 donkey anti-mouse (A21203) and Alexa Fluor^®^ 594 donkey anti-rabbit (A21207) from (Life Technologies).

### Expression constructs, siRNA and transfections

U20S, HeLa or HEK293T cells were transfected with either control esiRNA (MISSION^®^ siRNA targeting EGFP, Sigma-Aldrich) or COMMD4 esiRNA (Sigma-Aldrich) or alternatively control siRNA (MISSION^®^ siRNA Universal Negative Control #1, Sigma-Aldrich) or COMMD4 siRNA #2 (CCAUGUCCCUCUCAGCAGA[dT][dT] MISSION^®^ siRNA, Sigma-Aldrich), COMMD4 siRNA #3 (GUCUGCAGCCUACGCAUGA[dT][[dT] MISSION^®^ siRNA, Sigma-Aldrich) or COMMD4 siRNA #4 (GGUGUUCCAGGCCUGUGUG[dT][dT] MISSION^®^ siRNA, Sigma-Aldrich) and hSSB1 and ATM siRNA^20^, using RNAiMax (Life Technologies) as recommended by the manufacturer and samples were analysed 48–72 h post-transfection. HeLa, U2OS, HEK293 or HEK293T cells were transfected with a COMMD4 siRNA-resistant plasmid^18^ using FuGENE^®^ HD (Promega) as per manufacturer’s instructions and samples were analysed 24 h post-transfection. H2B and H2B S14E mutant with a C-terminal FLAG tag were cloned into the mammalian expression vector pCDNA3.1/Zeo (−) and were purchased from GenScript. The plasmids were transfected into HeLa or HEK293T cells using FuGENE^®^ HD and samples were analysed 24 h post-transfection. COMMD4 with an N-terminal His tag, cloned into the expression vector pET-28a(+), was purchased from GenScript.

### Subcellular fractionation and immunoblotting

Cells were separated into subcellular fractions using the subcellular protein fractionation kit for cultured cells (Thermo Scientific), according to the manufacturer’s instructions. Total cell lysates were prepared using ice-cold NP40 buffer (20 mM HEPES pH 8, 150 mM KCl, 10 mM MgCl_2_, 0.5 mM EDTA, 0.2% NP40, 0.5 mM DTT, 5% glycerol, 1X protease inhibitor cocktail, 1X phosphatase inhibitor cocktail and 1X Pierce Universal Nuclease for cell lysis (Thermo Fisher)) and lysed by sonication^48,49^. Protein concentrations were estimated using a Bicinchoninic acid kit for protein determination (Sigma-Aldrich) and subsequently 5 μg of the fractions and/or 15 μg of total cell lysate were separated on 4–12% Bis-Tris Plus Bolt precast gels (Life Technologies), immunoblotted with the antibodies shown and imaged with an Odyssey infra-red imaging system (LI-COR).

### Immunofluorescence microscopy

U20S and HeLa cells transfected with control or COMMD4 siRNA were grown in optical glass bottom 96 well plates (Cellvis) or μ-slide 8 well glass bottom chamber slides (ibidi). Following IR treatment, cells were pre-extracted for 5 min with ice-cold extraction buffer (20 mM HEPES (pH 8), 20 mM NaCl, 5 mM MgCl_2_, 1 mM ATP, 0.5% NP40), to remove the soluble proteins and processed as previously described^22^. Images were taken using a Delta Vision PDV microscope, ×600/1.42 or ×100/1.42 Oil objective (Applied Precision, Inc) or the OMX Blaze deconvolution SIM super-resolution microscope, ×60/1.42 two Oil objective (Applied Precision, Inc). All immunofluorescence figures were assembled using Adobe Photoshop CS6. High content imaging was performed using the IN Cell Analyzer 2200 Imaging System (GE Healthcare Life Sciences). Images were analysed using the IN Cell Investigator software (GE Healthcare Life Sciences) with a minimum of 50 nuclei quantified per independent experiment.

### Colony forming assays

For colony forming assays, following siRNA-depletion, 500 cells for each condition were plated in well of a 6-well plate, treated with the DNA damaging agent the following day and allowed to recover for 8–10 days^20,50^. Each condition was plated in triplicate and repeated three independent times. Data are represented as means and SD.

### Neutral comet assay

The neutral comet assay was carried out using U2OS cells that were treated or mock treated with 6 Gy of IR. Briefly, ± treatment, cells were embedded in agarose followed and electrophoresed. Cells were lysed as previous (10 mM Tris, pH 10, 2.5 M NaCl, 100 mM EDTA and 1% Triton X100) and singular cells were stained with Sybr Green (Thermo Fisher Scientific), with 50 cells chosen per condition for analysis and ImageJ was used to analyse the relative olive tail moment^48^.

### Homologous recombination assays

HR was assessed by determining the frequency of reconstitution of the green fluorescent protein reporter gene (pDRGFP) stably expressed in MCF7 cells^51^. MCF7 DRGFP cells were initially depleted of hSSB1 or COMMD4 and 48 h later *I-Sce1* was transiently expressed from the pCBSCE expression vector. Flow cytometry (Gallios Flow Cytometer, Beckman Coulter Life Sciences) was subsequently performed to determine the percentage of GFP positive cells and analysed using the Kaluza software (Beckman, Lane Cove NSW, Australia).

### Non-homologous end-joining assay

The pEGFP-N3 vector (Clontech) was digested with HindIII (New England BioLabs) for 6 h to cleave between to GFP coding region and promoter. The plasmid was subsequently separated by agarose gel electrophoresis and then gel purified using a gel clean-up kit (Promega). HEK293T cells transfected with either control or COMMD4 siRNA were subsequently transfected with either the uncut pEGFP-N3 or HindIII linearised plasmid, using FuGENE^®^ HD (Promega) and incubated for 48 h prior to harvest. Flow cytometry was performed to determine the percentage of GFP positive cells^29^.

### Analysis of NHEJ and HR function using an in vivo fluorescence-based reporter

U20S cells stably expressing the DNA repair reporter were transfected with control and COMMD4 siRNA, as previously described. In total, 48 h post-transfection, the cells were transfected with both the *I-Sce1* and exogenous donor plasmids. In total, 48 h post-transfection of the plasmids, cells were trypsinised and flow cytometry was performed to determine the percentage of GFP positive and mCherry positive cells^31^.

### Co-immunoprecipitations

Assays for co-immunoprecipitations were performed at 4 °C from HEK293T, HEK293 or HeLa cells transiently expressing the full-length COMMD4-FLAG plasmid and cells were lysed with ice-cold NP40 buffer. The plasmid pCMV6-AC-3DDK (FLAG) was used as the negative control and proteins were captured using anti-FLAG M2 Dynabeads (Sigma-Aldrich) and washed three times in NP40 buffer prior to analysis.

### Direct interaction between COMMD4 and hSSB1

In total, 300 ng of recombinant COMMD4, purified from the pET-28a expression vector, was incubated with either 500 ng of WT hSSB1^21^, T117E^20^ or S134E^33^ for 1 h at 4 °C in NP40 buffer. COMMD4 antibody bound to Protein A Dynabeads (Thermo Fisher Scientific) was used to pull-out the complex. The beads were washed three times with NP40 buffer, resuspended in 4X Laemmli sample buffer (250 mM Tris pH 6.8, 8% SDS, 4% glycerol, 0.02% bromophenol blue, 8% β-mercaptoethanol) and immunoblotted with anti-COMMD4 and anti-hSSB1 antibodies.

### Chromatin immunoprecipitation

ChIP assays were performed using the SimpleChIP^®^ Plus Sonication Chromatin IP Kit (Cell Signalling) in DRGFP cells^51^, according the manufacturer’s procedure and additionally using the protocol previously described^52^. Briefly, COMMD4-FLAG was overexpressed in DRGFP cells and 16 h later, cells were transfected with *I-Sce1*. In total, 12–24 h post-transfection with *I-Sce1*, ~2–5 × 10^7^ DR-GFP cells with and without *I-Sce1* induction were fixed with formaldehyde to cross-link the DNA and proteins and chromatin was sheared by sonication. FLAG antibody was used to immunoprecipitate COMMD4, the protein-DNA cross-links were reversed and DNA was purified and analysed by qPCR using the primers previously described^20,34^.

### Direct interaction between COMMD4 and H2B

FLAG-tagged COMMD4 was purified from stable HEK293 cells expressing COMMD4-FLAG, while we purchased recombinant H2B (NEB) and ATM (Merck). Lysates were incubated for 1 h at 4 °C either with protein G Dynabeads (Thermo Fisher Scientific) bound with FLAG antibody or normal mouse IgG. Following the incubation, the beads were washed with high salt buffer (20 mM HEPES pH 8, 500 mM KCl, 10 mM MgCl_2_, 0.5 mM EDTA, 0.2% NP40, 5% glycerol) followed by a wash with 250 mM Lithium chloride to remove the non-specific interactors. In total, 700 ng of H2B was pre-incubated with 35 ng of recombinant active ATM for 30 min at 37 °C in ATM kinase buffer (10 mM Hepes pH 7.5, 50 mM β-glycerophosphate, 50 mM NaCl, 10 mM MgCl_2_, 10 mM MnCl_2_, 1 mM DTT, 500 μM ATP) with and without the ATM inhibitor, KU-55933 (Selleckchem). Following the pre-incubation, H2B was added to COMMD4-FLAG for 10 min in the absence or presence of ATM. The beads were subsequently washed several times with high and medium salt buffer (20 mM HEPES pH 8, 250 mM KCl, 10 mM MgCl_2_, 0.5 mM EDTA, 0.2% NP40, 5% glycerol), and resuspended in 4X Laemmli sample buffer and immunoblotted with anti-H2B and anti-FLAG antibodies.

### Structure of histone H2A-H2B

The structure of the histone H2A-H2B dimer (PDB: 2RVQ)^53^ was downloaded from the protein data bank.

### Mapping the interaction site of H2B and COMMD4

A peptide-mapping strategy was used to determine the exact site of interaction between H2B and COMMD4. H2B was reduced to a series of 20 partially overlapping peptides containing a C-terminal Biotin tag (Supplementary Table 1) and was purchased from GenScript. In total, 300 ng of recombinant COMMD4, purified from the pET-28a expression vector, was incubated with 1.2 μg of each peptide in NP40 buffer for 1 h at 4 °C. Proteins were captured using Streptavidin Dynabeads (Sigma-Aldrich), washed three times in NP40 buffer, resuspended in 4X Leammli sample buffer and immunoblotted with the anti-COMMD4 antibody.

### Mapping the interaction site of RNF20/40 and H2B

In total, 700 ng of recombinant active human RNF20 or RNF40 (University of Dundee) was incubated with 1.2 μg of each peptide in NP40 buffer for 1 h at 4 °C. Proteins were captured using Streptavidin Dynabeads (Sigma-Aldrich), washed three times in NP40 buffer, resuspended in 4X Leammli sample buffer and immunoblotted with the anti-RNF20 or RNF40 antibodies.

### In vitro ubiquitination assay

FLAG-tagged COMMD4 was purified from stable HEK293 cells expressing COMMD4-FLAG, while we purchased recombinant H2B (Cell Signalling), human E1 Ubiquitin Activating Enzyme Protein (Abcam), human Ube2B E2 protein (Abcam), human active RNF20 and RNF40 and human Ubiquitin protein (Abcam). FLAG-tagged COMMD4 was purified as described earlier. In total, 50 ng of E1, 300 ng of E2 and 5 μg of ubiquitin was added to 3.5 μl of 10X Ubiquitination buffer (50 mM Tris pH 8, 5 mM MgCl_2_, 0.1% Tween 20, 1 mM DTT). To the reaction, 500 μM ATP, FLAG-tagged COMMD4 on Protein G Dynabeads and 100 ng of H2B was added, the reaction was made up to 48 μl with water and 200 ng of RNF20/40 was added to make up the final reaction volume to 50 μl. The reaction was then incubated for 30 min at 4 °C and following the incubation, resuspended in 4X Laemmli sample buffer and immunoblotted with the antibodies indicated.

### Direct interaction between COMMD4 and H2A or H2B

In total, 300 ng of recombinant COMMD4, purified from the pET-28a expression vector, was incubated with either 300 ng of H2A (NEB) or H2B (NEB) for 1 h at 4 °C in NP40 buffer. H2A or H2B bound to Protein A Dynabeads (Thermo Fisher Scientific) was used to pull-out the complex. The beads were subsequently washed several times with high salt buffer, resuspended in 4X Laemmli sample buffer and immunoblotted with anti-COMMD4 and anti-H2A/H2B antibodies.

### In vitro binding of H2A, H2B and COMMD4

In total, 300 ng of H2A was pre-incubated with 2 μl of recombinant active CK2 (500,000 units/ml, NEB) for 1 h at 30 °C in kinase buffer. Following the pre-incubation, 300 ng of H2B was added at 4 °C for 30 min, followed by the addition of 300 ng of recombinant COMMD4 for 30 min at 4 °C. H2B or COMMD4 bound to protein A Dynabeads (Thermo Fisher Scientific) were used to pull-out the bound complexes. The beads were subsequently washed several times with high and medium salt buffer, resuspended in 4X Laemmli sample buffer and immunoblotted with the antibodies indicated.

### In vitro binding of COMMD4 and nucleosomes

In total, 780 ng of human native nucleosomes (Merck) were pre-incubated with and without 2 μl of recombinant active CK2 (500,000 units/ml, NEB) and 35 ng of recombinant active ATM, for 1 h at 30 °C in kinase buffer. Following the pre-incubation, 300 ng of recombinant COMMD4 was added for 30 min at 4 °C. COMMD4 bound to protein A Dynabeads (Thermo Fisher Scientific) were used to pull-out the bound complexes. The beads were subsequently washed several times with high and medium salt buffer, resuspended in 4X Laemmli sample buffer and immunoblotted with the antibodies indicated.

### Statistics and reproducibility

Data are presented as means and SD or SEM where shown, from ≥3 independent experiments. Statistical analyses were performed using a two-tailed non-paired Student’s *t* test. The level of significance was set at ns (not significant) *p* > 0.05, *p < 0.05 and **p < 0.005. No statistical methods were used to predetermine sample size.

### Reporting summary

Further information on research design is available in the Nature Research Reporting Summary linked to this article.

## Supplementary information

Supplementary Information

Description of Additional Supplementary Files

Supplementary Data 1

Reporting Summary

## Data Availability

Source data underlying figures is presented in Supplementary Data 1. Plasmids and other relevant data are available from the corresponding authors upon reasonable request.
