# Supplementary material for: COMMD4 functions with the histone H2A-H2B dimer for the timely repair of DNA double-strand breaks
Source: Commun Biol. 2021 Apr 19;4:484. doi: 10.1038/s42003-021-01998-2 (PMC8055684; doi:10.1038/s42003-021-01998-2)
Supplement: Supplementary file 1 — Supplementary Information [file 42003_2021_1998_MOESM1_ESM.pdf]

# Supplementary Information

# COMMD4 functions with the histone H2A-H2B dimer for the timely repair of DNA double-strand breaks

Amila Suraweera, Neha S. Gandhi, Sam Beard, Joshua T. Burgess, Laura V. Croft, Emma Bolderson, Ali Naqi, Nicholas W. Ashton, Mark N. Adams, Kienan Savage, Shu-Dong Zhang, Kenneth J. O'Byrne, Derek J. Richard.

## Supplementary Methods

### hSSB1 immunoprecipitations

hSSB1 immunoprecipitations were performed as previously described [1] using ice-cold NP40 buffer and performed at 4 °C from HEK293T cells. hSSB1 antibody or IgG was used to immunoprecipitate the bound proteins, which were captured using Protein G Dynabeads and washed three times in NP40 buffer prior to analysis.

### Direct interaction between COMMD4 and RNF20 or RNF40

FLAG-tagged COMMD4 was purified from stable HEK293 cells expressing COMMD4-FLAG, while we purchased recombinant human active RNF20 (Sigma-Aldrich) and recombinant active RNF40 (Genbank NM_014771, University of Dundee). Purified COMMD4-FLAG was incubated with either 1 μg of RNF20 or RNF40 for 1 h at 4 °C in NP40 buffer. The beads were subsequently washed several times with high salt buffer, resuspended in 4X Laemmli sample buffer and immunoblotted with anti-FLAG, RNF20 and RNF40 antibodies.

***In vitro* binding of RNF40 and H2B peptides**

1.2 μg of H2B peptides 1 and 7 were pre-incubated with or without 35 ng of recombinant active ATM for 30 min at 37 °C in ATM kinase buffer. Following the pre-incubation, 700 ng of recombinant active human RNF40 was added for 1 h at 4 °C. The beads were subsequently washed several times with high and medium salt buffer and resuspended in 4X Laemmli sample buffer and immunoblotted with anti-RNF40 antibody.

**COMMD4 mutants**

Wild-type (WT) and COMMD4 mutants (1BD and 1DB2) (Supplementary Table 2) were cloned into the mammalian expression vector pCDNA3.1/Zeo (-) and were purchased from GenScript. HEK293T cells were initially transfected with COMMD4 siRNA #2 as previously described in this manuscript. 48 h post-transfection, COMMD4 mutants were overexpressed in these cells using FuGENE® HD and 24 h post-transfection of the plasmid cells were harvested.

## Supplementary Figures

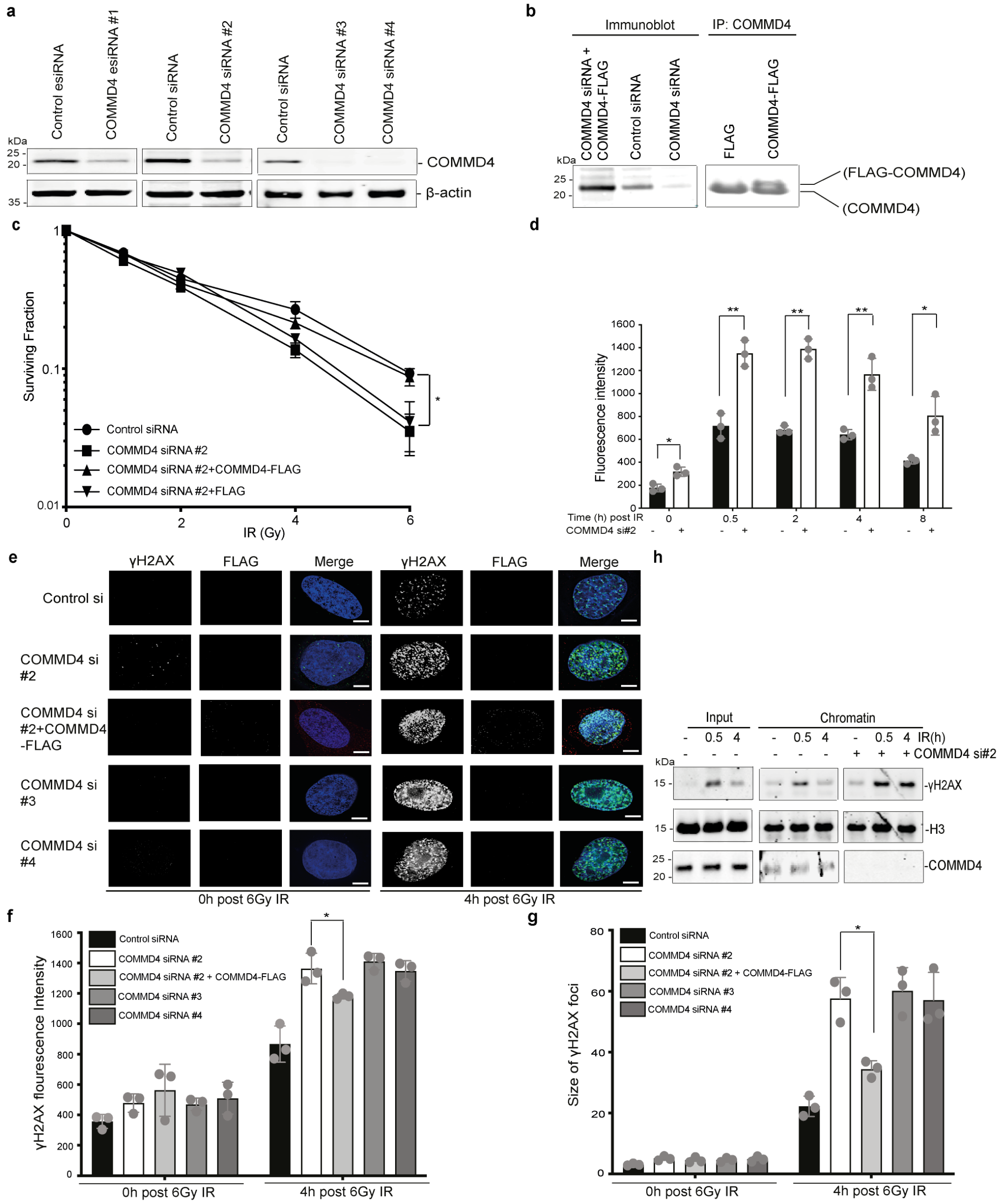


**Supplementary Fig. 1**: **COMMD4 is required for survival following DNA damage**. **a**. Immunoblot showing the depletion of COMMD4 using control siRNA or COMMD4 esiRNA (#1), siRNA #2, siRNA #3 and siRNA #4. β-actin shows the loading. **b**. Immunoblot and immunoprecipitation of COMMD4 from FLAG or siRNA-resistant COMMD4-FLAG expressing HeLa cells. **c**. A clonogenic assay demonstrating the correction of the IR defect in cells depleted of COMMD4 (siRNA #2) using a COMMD4 siRNA-resistant plasmid. AT5; cells from an A-T patient. **d**. Plot of the γH2AX intensity for Fig. **1c** using high content imaging. **e**. Immunofluorescence showing IR-induced foci at 0 and 4 h after irradiation in control, COMMD4 siRNA and COMMD4-depleted cells overexpressing COMMD4-FLAG. **f** and **g**. Quantification of IR-induced foci for **e**. **h**. Subcellular fractionation followed by immunoblotting. H3 shows the loading. Scale bar denotes 5 µm. *; $P < 0.05$; **; $P < 0.005$. Error bars represent mean ± SD from three independent experiments.

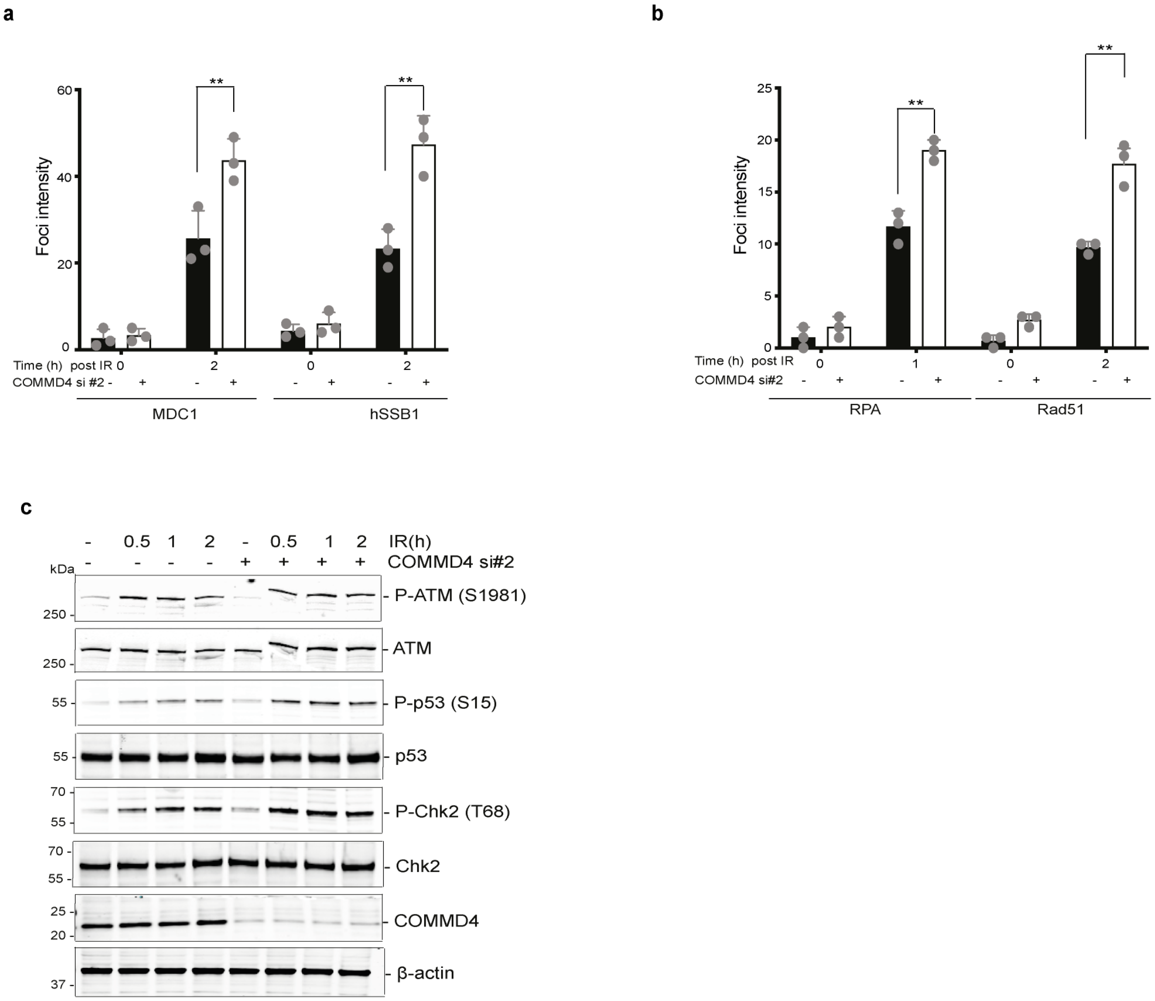


**Supplementary Fig. 2**: **Genomic instability in COMMD4-depleted cells and complementation of the defect**. **a**. Quantification of MDC1 and hSSB1 foci intensity for Fig. **1f**. **b**. Quantification of RPA and Rad51 foci intensity for Fig. **1g** and **h**. *; $P < 0.05$ and **; $P < 0.005$. Error bars represent mean ± SD from three independent experiments where 50 cells were quantified per condition. **c**. Immunoblot of a time course for control and cells depleted of COMMD4, after treatment with 6 Gy of IR followed by recovery at time points shown. β-actin was used as the loading control.

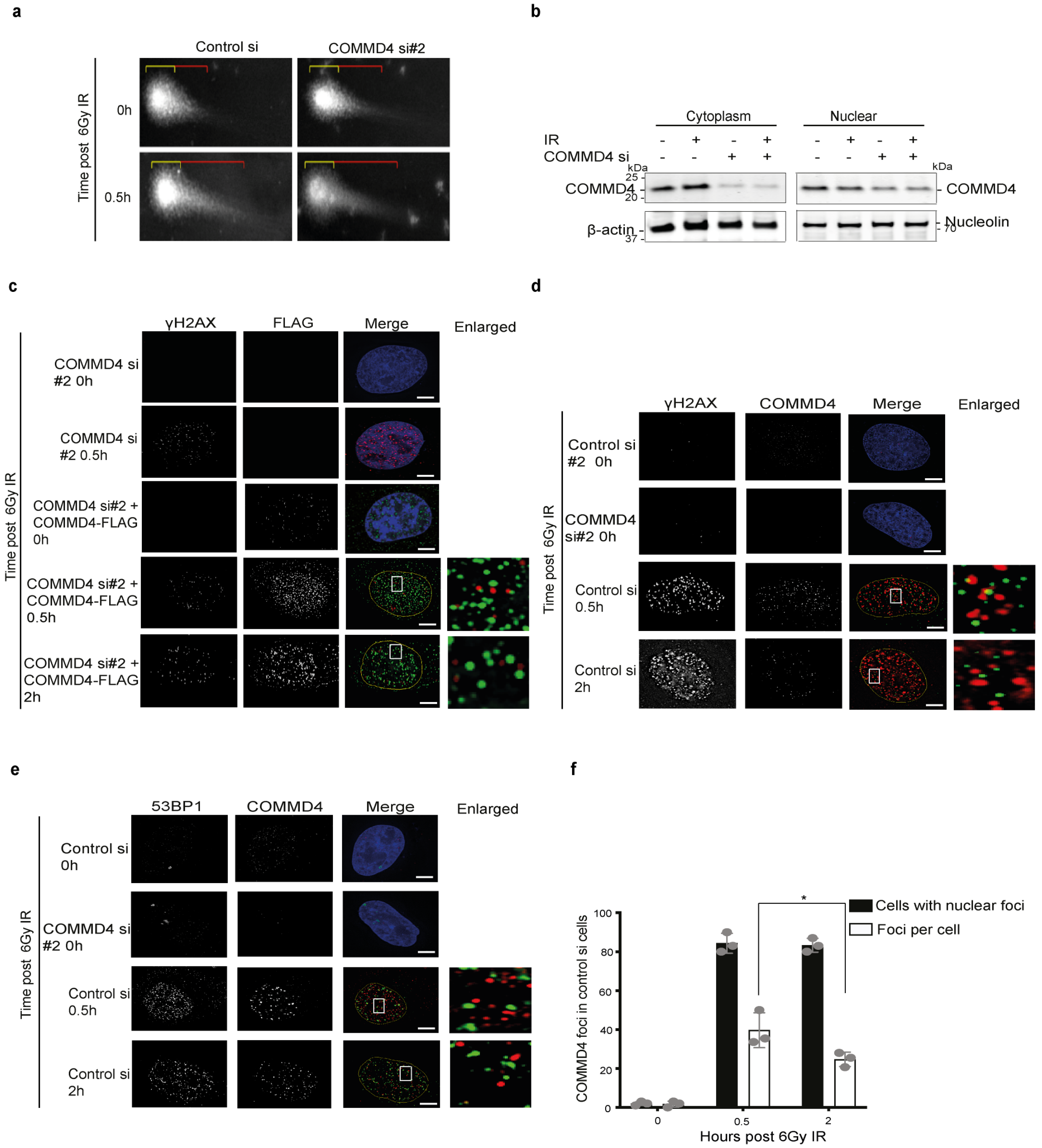


**Supplementary Fig. 3: Repair of DNA double-strand breaks and localisation of COMMD4**. **a**. Comet assay showing the relative comet tail moment in COMMD4-deficient and control cells at 0 h and 0.5 h irradiation. **b**. Subcellular fractionation on control and COMMD4-deficient cells pre and 4 h post-IR. Fractions were subjected to immunoblotting and probed with the indicated antibodies. Actin and nucleolin show the loading controls for the cytoplasmic and soluble nuclear fractions respectively. **c**. Immunofluorescence in COMMD4-depleted cells with and without overexpression with COMMD4-FLAG followed by 2 Gy irradiation and recovery at time points shown. **d** and **e**. Immunofluorescence in control and COMMD4-depleted cells following recovery after irradiation. **f**. Quantification of the COMMD4 foci for **d** and **e**. *; $P < 0.05$. Error bars represent mean ± SD from three independent experiments where 50 cells were quantified per condition. Scale bar denotes 5 μm.

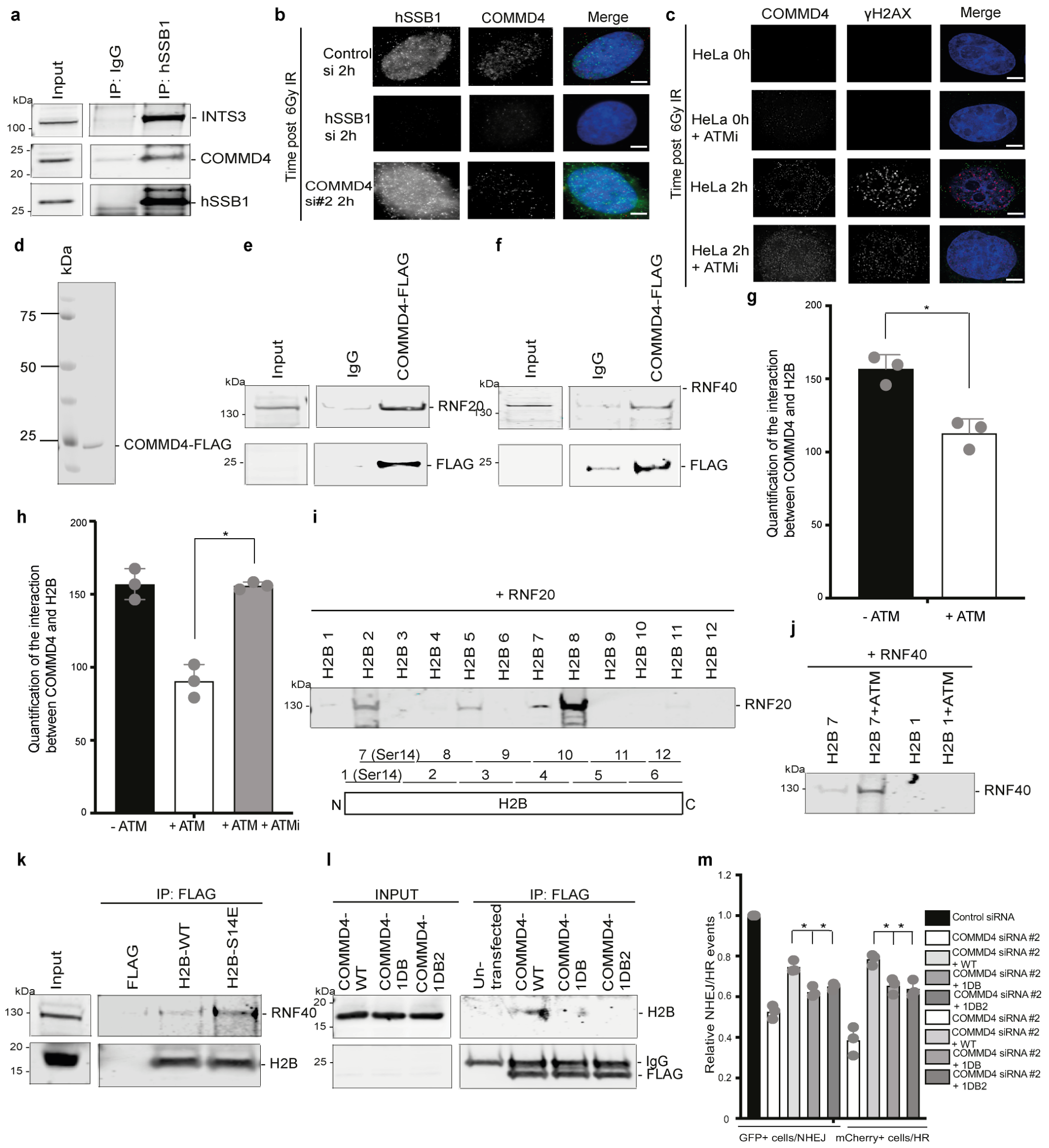


**Supplementary Fig. 4**: **Interaction between COMMD4 and hSSB1, H2B, RNF20 and RNF40**. **a**. hSSB1 was immunoprecipitated from cells and the co-eluting proteins were immunoblotted with the antibodies shown. INTS3 is the positive control for hSSB1 immunoprecipitation and IgG the negative control. **b**. Immunofluorescence showing hSSB1 and COMMD4 staining 2 h after irradiation. **c**. γH2AX and COMMD4 staining in HeLa cells 2 h after irradiation, +/- ATM inhibitor (ATMi). DAPI shows the nucleus. Scale bar denotes 5 µm. **d**. A coomassie gel demonstrating the purification of COMMD4-FLAG from HEK293 cells stably expressing COMMD4-FLAG. **e** and **f**. A direct interaction between COMMD4 and RNF20 or RNF40 with recombinant COMMD4-FLAG and recombinant RNF20 or 40. **g**. Quantification of Fig. **3c**, a direct interaction between COMMD4 and H2B with and without recombinant ATM. **h**. Quantification of Fig. **3d**, a direct interaction between COMMD4 and H2B, +/- recombinant ATM and ATM inhibitor (ATMi). **i**. Direct interaction between RNF20 and H2B demonstrating the specific binding regions within H2B. Schematic below shows the peptide regions within H2B and the Ser14 site. **j**. Direct interaction between RNF40 and H2B peptides 1 and 7 +/- recombinant ATM. **k**. FLAG and FLAG-tagged H2B WT, and S14E mutant were immunoprecipitated and the co-eluting proteins were immunoblotted with RNF40 and H2B. **l.** COMMD4 (WT) and FLAG-tagged COMMD4 mutants (1DB and 1DB2) were immunoprecipitated and the co-eluting proteins were immunoblotted with FLAG and H2B. IgG shows loading. **m.** Plot of relative NHEJ/HR with COMMD4 WT and mutants. *****; $P < 0.05$. Error bars represent mean ± SD from three independent experiments.

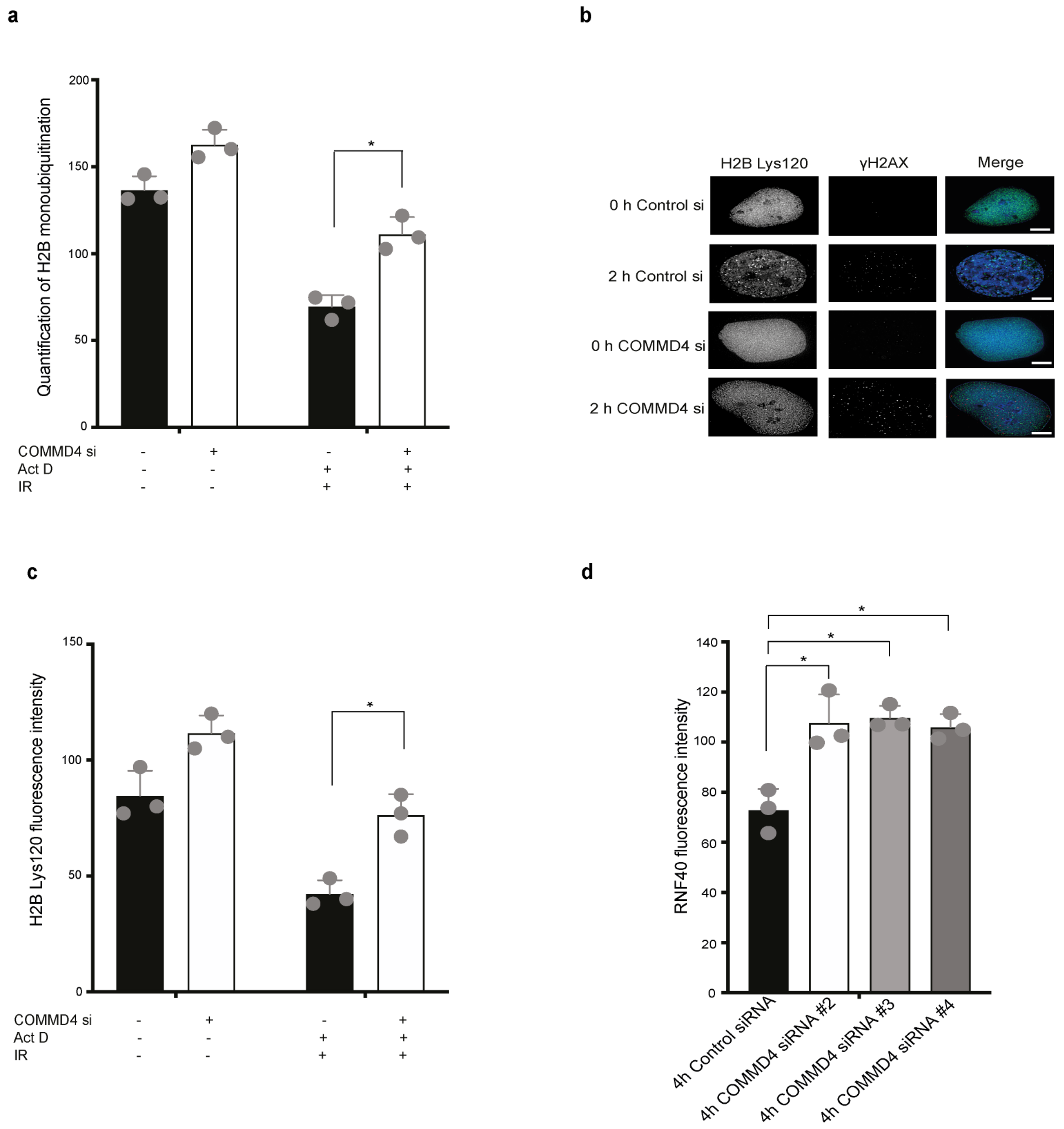


**Supplementary Fig. 5: ATM-dependent monoubiquitination of H2B.** a. Quantification of Fig. **4a**, an immunoblot showing H2B monoubiquitination in control and COMMD4-depleted cells with 5 µg/ml of Actinomycin D (Act D) and +/- irradiation. **b**. Immunofluorescence showing H2B monoubiquitinated protein (H2B Lys120) in control and COMMD4-depleted cells with actinomycin D, +/- irradiation. **c**. Quantification of H2B 120 levels for **b**. **d**. Quantification of the RNF40 intensity in control and COMMD4 depleted cells at 4 h post-IR for Fig. **4b**. *; $P < 0.05$. Error bars represent mean ±SD from three independent experiments. 50 cells were quantified per immunofluorescence condition. Scale bar denotes 5 µm.

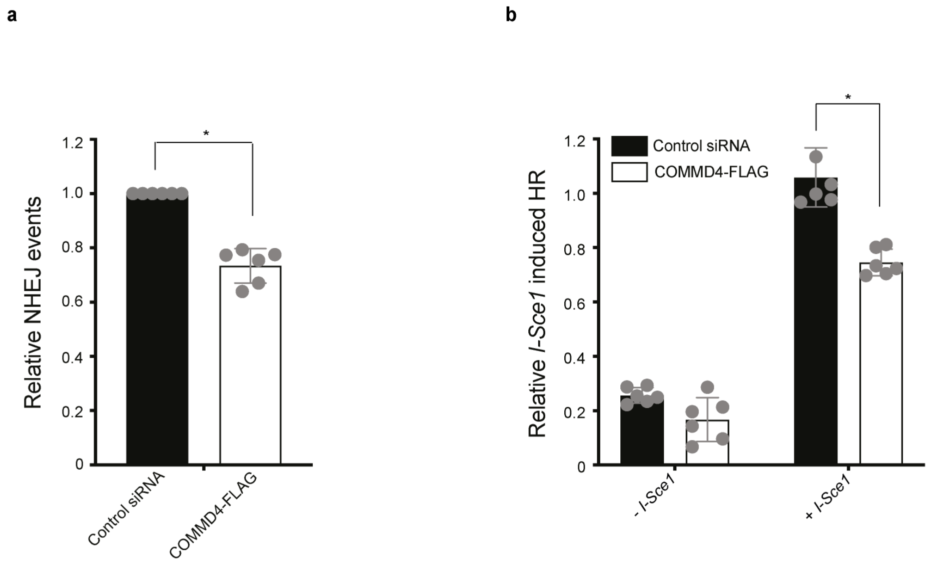


**Supplementary Fig. 6: Repair of DSBs in COMMD4-depleted cells. a and b**. Plot of the relative NHEJ and relative HR events, respectively, in control and cells overexpressing COMMD4-FLAG transiently (without the depletion of COMMD4). *; $P < 0.05$ from five independent experiments. Error bars represent mean ± SD.

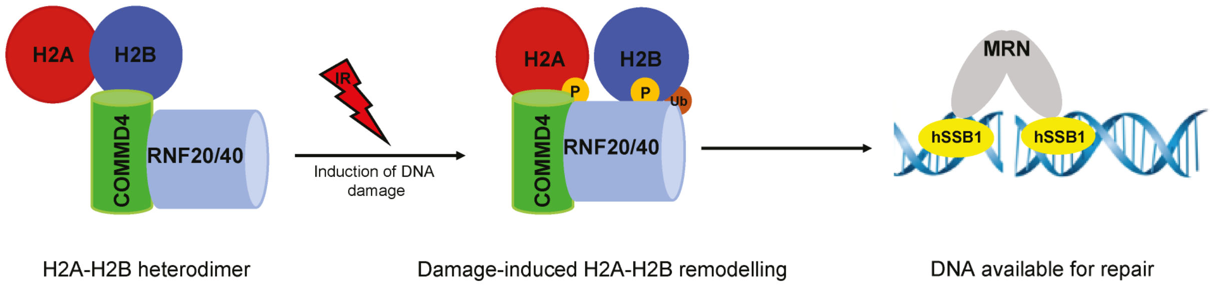


**Supplementary Fig. 7: COMMD4 and the histone H2A-H2B dimer**. A schematic depicting how COMMD4 functions in DNA damage-induced H2A-H2B remodeling. P; shows the phosphorylation of Ser14 of H2B or at Tyr57 of H2A; Ub; monoubiquitination of H2B by the RNF20/40 complex.

## Supplementary Tables

**Supplementary Table 1: H2B peptide sequences**

| Amino Acid Number | Sequence |
|---|---|
| 1. 1-20 | MPEPSKSAPAPKKGSKKAIT{LYS(BIOTIN-AHX)} |
| 2. 21-40 | KAQKKDGKKRKRSRKESYSI{LYS(BIOTIN-AHX)} |
| 3. 41-60 | YVYKVLKQVHPDTGISSKAM{LYS(BIOTIN-AHX)} |
| 4. 61-80 | GIMNSFVNDIFERIAGEASR{LYS(BIOTIN-AHX)} |
| 5. 81-100 | LAHYNKRSTITSREIQTAVR{LYS(BIOTIN-AHX)} |
| 6. 101-126 | LLLPGELAKHAVSEGTKAVTKYTSSK{LYS(BIOTIN-AHX)} |
| 7. 5-24 | SKSAPAPKKGSKKAITKAQK{LYS(BIOTIN-AHX)} |
| 8. 25-44 | KDGKKRKRSRKESYSIYVYK{LYS(BIOTIN-AHX)} |
| 9. 45-64 | VLKQVHPDTGISSKAMGIMN{LYS(BIOTIN-AHX)} |
| 10. 65-84 | SFVNDIFERIAGEASRLAHY{LYS(BIOTIN-AHX)} |
| 11. 85-104 | NKRSTITSREIQTAVRLLLP{LYS(BIOTIN-AHX)} |
| 12. 105-126 | GELAKHAVSEGTKAVTKYTSSK{LYS(BIOTIN-AHX)} |

**Supplementary Table 2: Sequence of COMMD4 mutants**

| Plasmid name | COMMD4 sequence |
|---|---|
| 1. WT | COMMD4-FLAG |
| 2. 1DB | COMMD4-FLAG with nucleotides: 52 C→G, 53 T→C, 59 A→C and 60 A→G |
| 3. 1DB2 | COMMD4-FLAG with nucleotides: 52 C→G, 53 T→C, 61 A→G, 62 T→C and 63 C→G |

**Supplementary References:**


1 Richard, D. J. *et al.* hSSB1 interacts directly with the MRN complex stimulating its recruitment to DNA double-strand breaks and its endo-nuclease activity. *Nucleic Acids Res* **39**, 3643-3651, doi:10.1093/nar/gkq1340 (2011).

## Uncropped blots

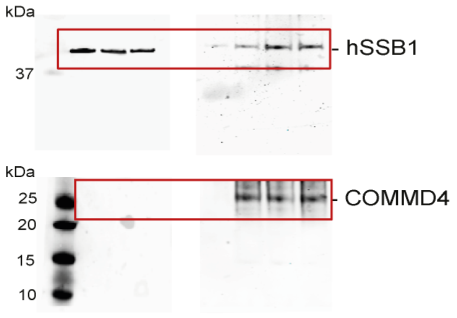


Uncropped blots related to Figure 2e

**Supplementary Fig. 8: Uncropped blots**. (related to Fig. 2).

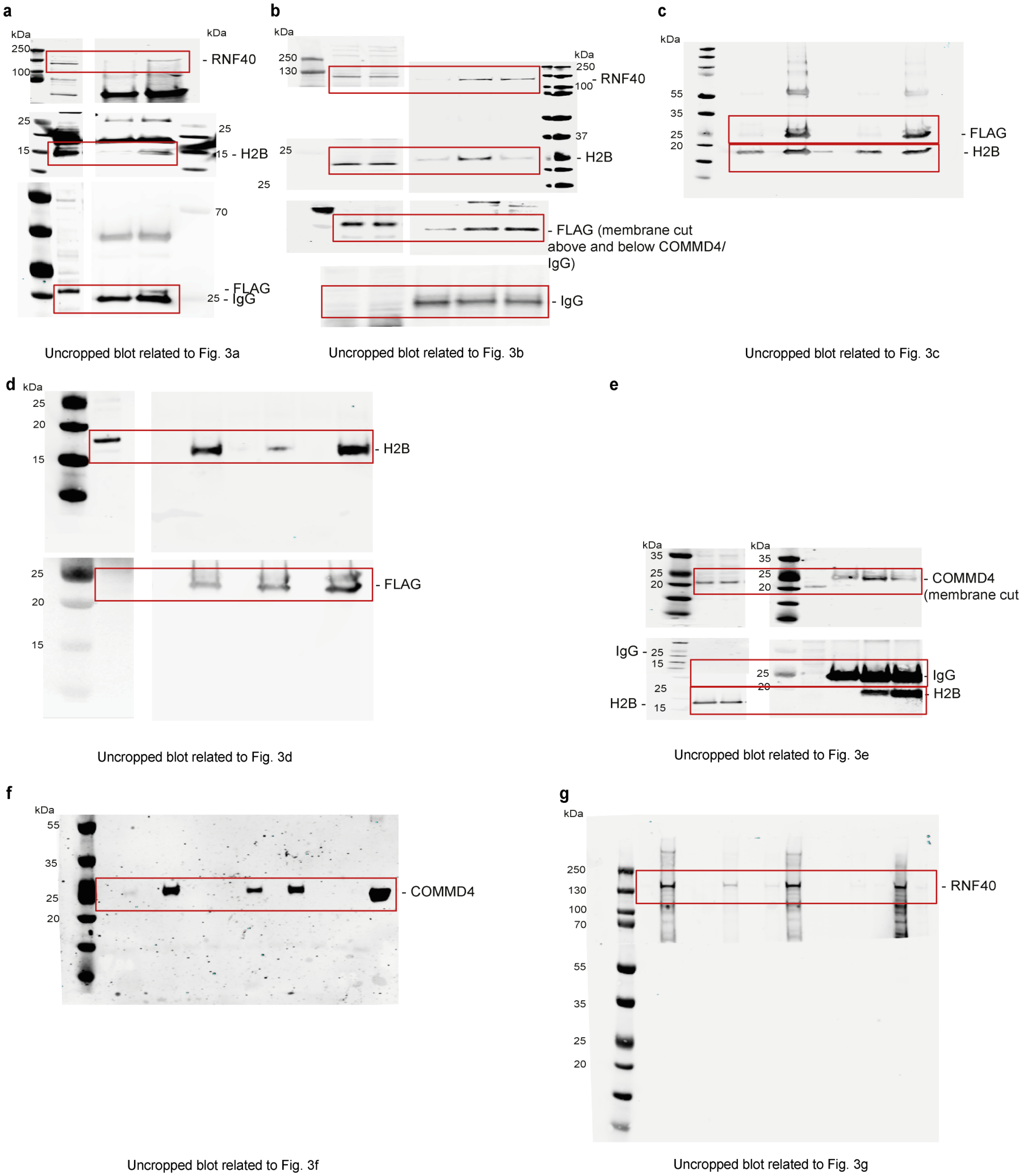


**Supplementary Fig. 9: Uncropped blots**. (related to Fig. 3).

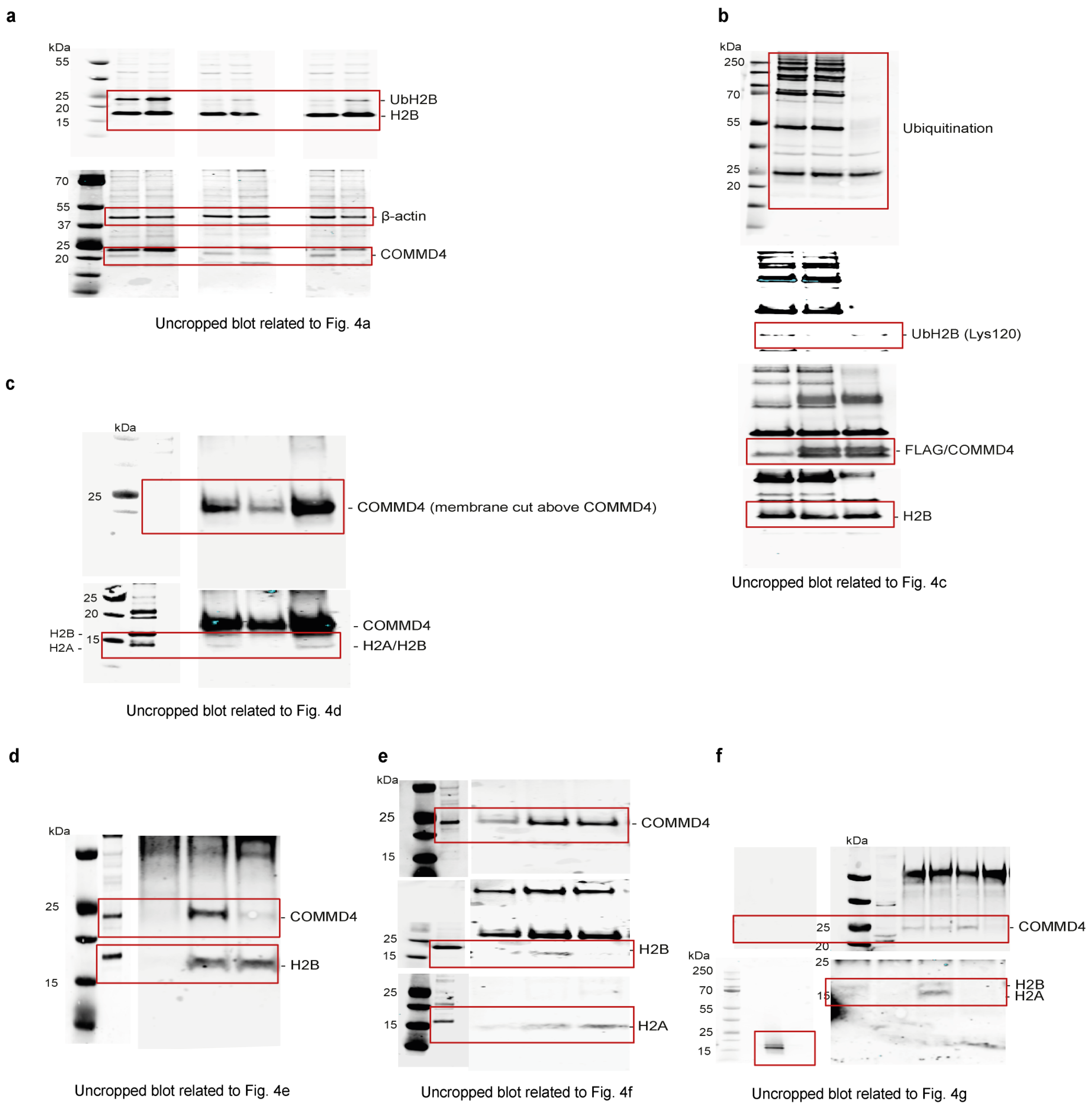


**Supplementary Fig. 10: Uncropped blots**. (related to Fig. 4).

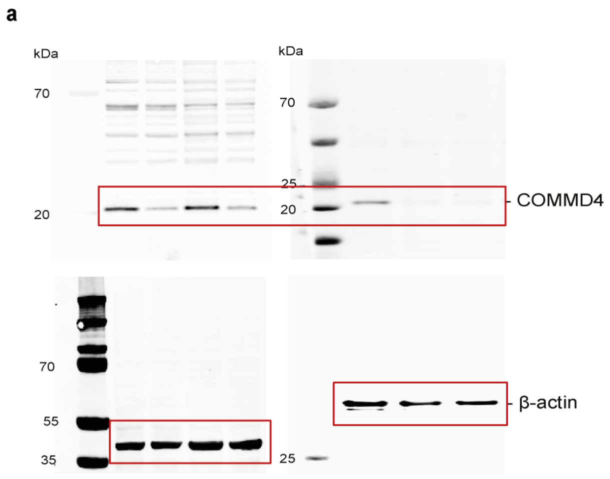


Uncropped blots related to Supplementary Fig. 1a

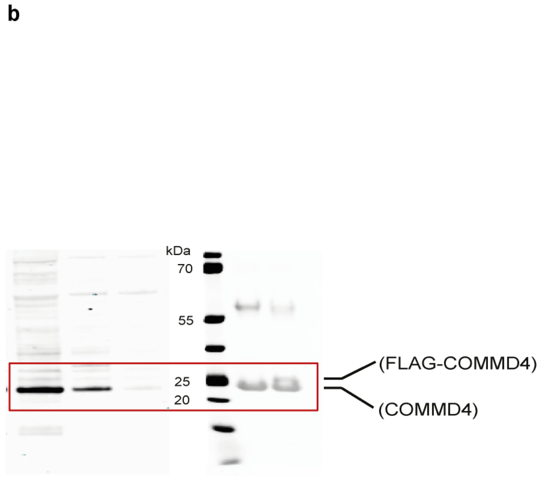


Uncropped blots related to Supplementary Fig. 1b

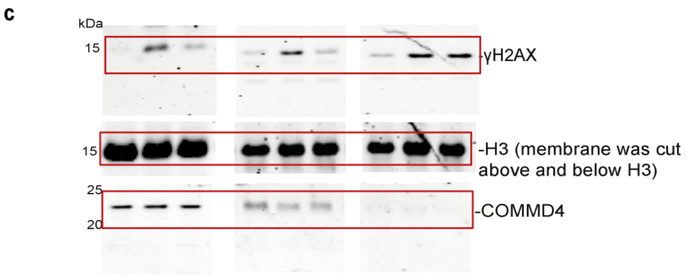


Uncropped blots related to Supplementary Fig. 1h

**Supplementary Fig. 11: Uncropped blots**. (related to Supplementary Fig. 1).

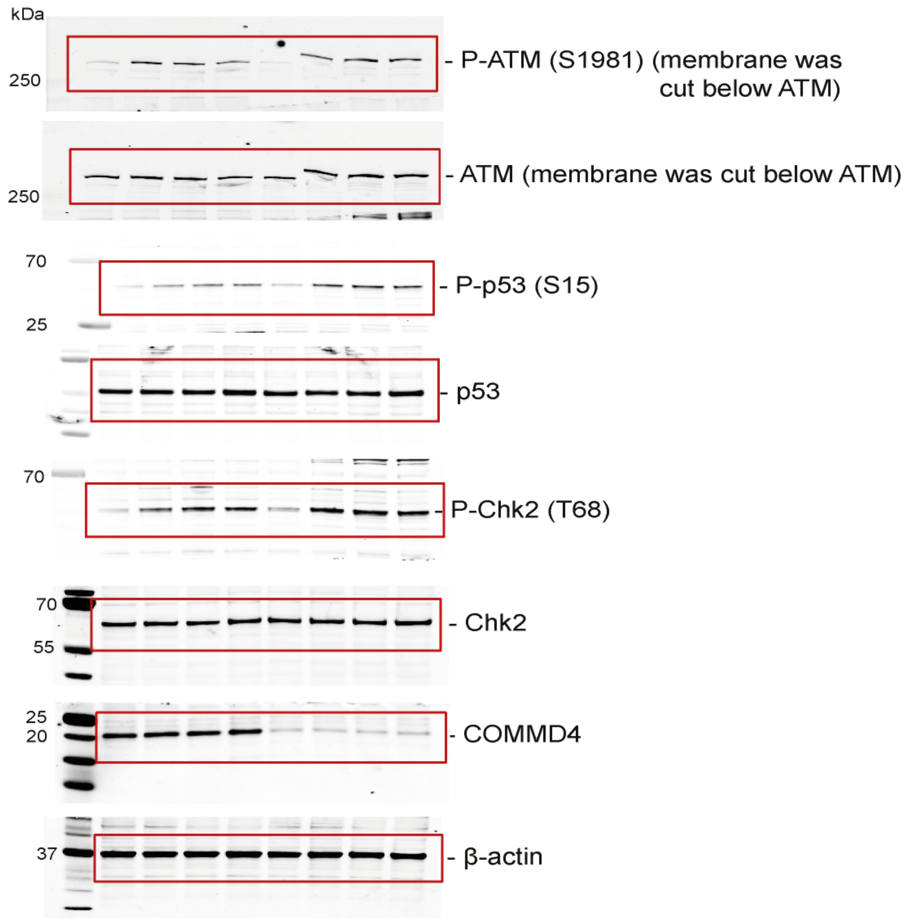


Uncropped blot related to Supplementary Fig. 2c

**Supplementary Fig. 12: Uncropped blots**. (related to Supplementary Fig. 2).

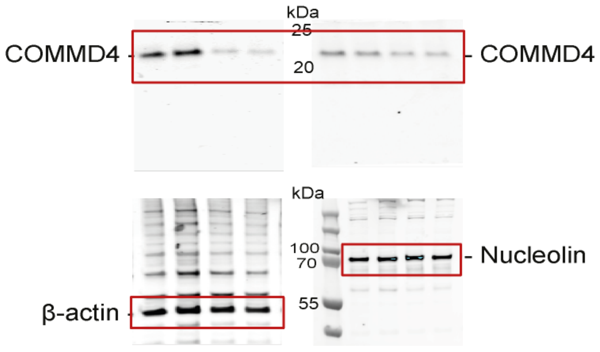


Uncropped blot related to Supplementary Fig. 3b

**Supplementary Fig. 13: Uncropped blots**. (related to Supplementary Fig. 3).

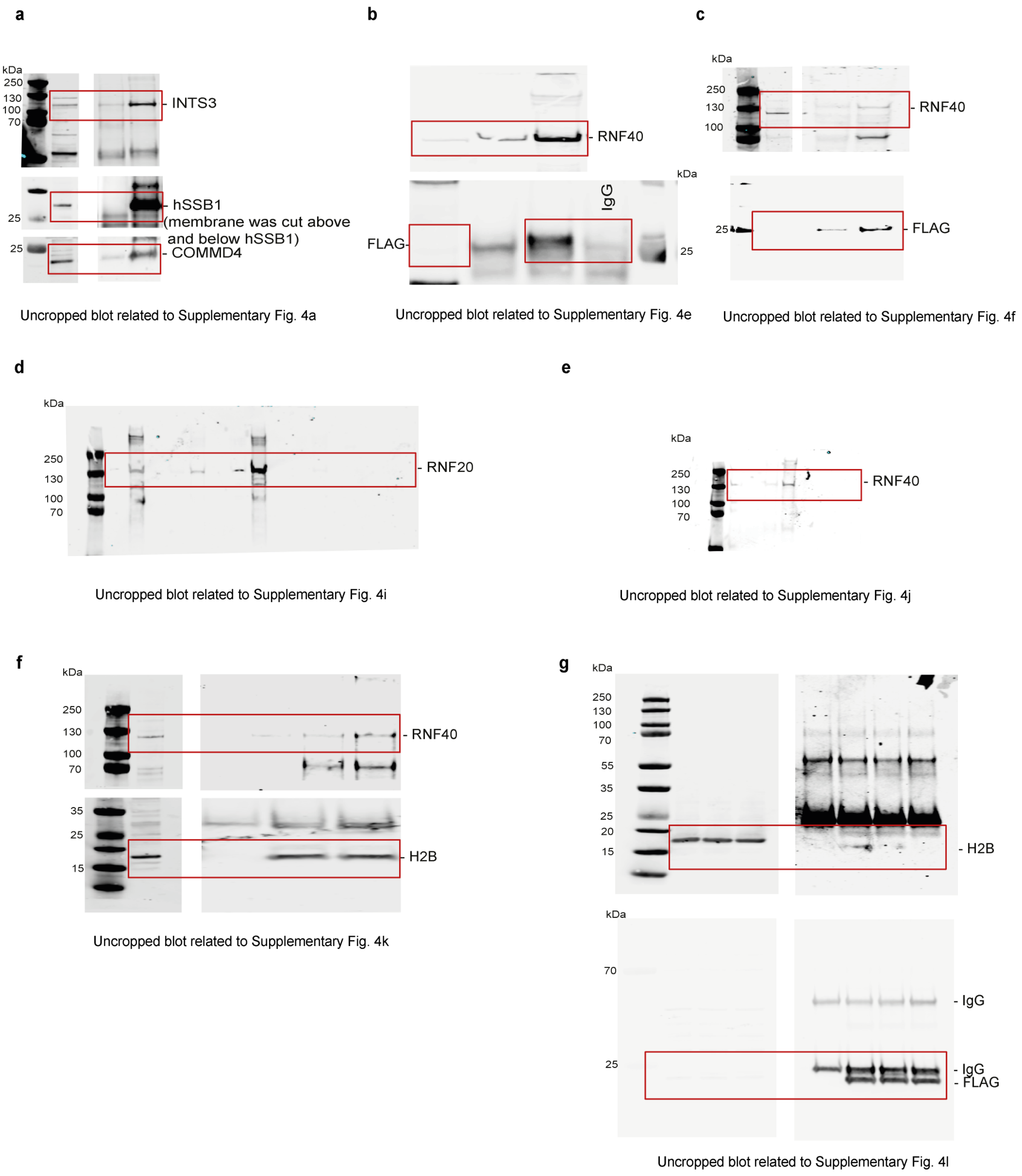


**Supplementary Fig. 14: Uncropped blots**. (related to Supplementary Fig. 4).
