# Supplementary material for: COMMD4 functions with the histone H2A-H2B dimer for the timely repair of DNA double-strand breaks
Source: Commun Biol. 2021 Apr 19;4:484. doi: 10.1038/s42003-021-01998-2 (PMC8055684; doi:10.1038/s42003-021-01998-2)
Supplement: Supplementary file 2 — Description of Additional Supplementary Files [file 42003_2021_1998_MOESM2_ESM.pdf]

Description of Additional Supplementary Files

File Name: Supplementary Data 1
Description: Source data
