# Supplementary material for: COMMD4 functions with the histone H2A-H2B dimer for the timely repair of DNA double-strand breaks
Source: Commun Biol. 2021 Apr 19;4:484. doi: 10.1038/s42003-021-01998-2 (PMC8055684; doi:10.1038/s42003-021-01998-2)
Supplement: Supplementary file 4 — Reporting Summary [file 42003_2021_1998_MOESM4_ESM.pdf]

nature research

Corresponding author(s): Amila Suraweera and Derek Richard

Last updated by author(s): Mar 12, 2021

Reporting Summary

Nature Research wishes to improve the reproducibility of the work that we publish. This form provides structure for consistency and transparency in reporting. For further information on Nature Research policies, see our Editorial Policies and the Editorial Policy Checklist.

Statistics

For all statistical analyses, confirm that the following items are present in the figure legend, table legend, main text, or Methods section.

n/a	Confirmed	
☐	☒	The exact sample size (*n*) for each experimental group/condition, given as a discrete number and unit of measurement
☐	☒	A statement on whether measurements were taken from distinct samples or whether the same sample was measured repeatedly
☐	☒	The statistical test(s) used AND whether they are one- or two-sided<br>*Only common tests should be described solely by name; describe more complex techniques in the Methods section.*
☒	☐	A description of all covariates tested
☐	☒	A description of any assumptions or corrections, such as tests of normality and adjustment for multiple comparisons
☐	☒	A full description of the statistical parameters including central tendency (e.g. means) or other basic estimates (e.g. regression coefficient) AND variation (e.g. standard deviation) or associated estimates of uncertainty (e.g. confidence intervals)
☐	☒	For null hypothesis testing, the test statistic (e.g. *F*, *t*, *r*) with confidence intervals, effect sizes, degrees of freedom and *P* value noted<br>*Give P values as exact values whenever suitable.*
☒	☐	For Bayesian analysis, information on the choice of priors and Markov chain Monte Carlo settings
☒	☐	For hierarchical and complex designs, identification of the appropriate level for tests and full reporting of outcomes
☒	☐	Estimates of effect sizes (e.g. Cohen's *d*, Pearson's *r*), indicating how they were calculated

Our web collection on statistics for biologists contains articles on many of the points above.

Software and code

Policy information about availability of computer code

Data collection	No software was used
Data analysis	GraphPad Prism V8 (statistical analysis), In Cell Investigator (immunofluorescence analysis), ImageJ (immunoblotting analysis), Adobe Photoshop CS6 and Adobe Illustrator CS6 (image analysis).

For manuscripts utilizing custom algorithms or software that are central to the research but not yet described in published literature, software must be made available to editors and reviewers. We strongly encourage code deposition in a community repository (e.g. GitHub). See the Nature Research guidelines for submitting code & software for further information.

Data

Policy information about availability of data

All manuscripts must include a data availability statement. This statement should provide the following information, where applicable:

- Accession codes, unique identifiers, or web links for publicly available datasets
- A list of figures that have associated raw data
- A description of any restrictions on data availability

The protein structures used are available from the Protein Data Bank (PBD code 2RVQ). Raw data for graphs and uncropped immunoblot blot images are available as Supplementary information.

Field-specific reporting

Please select the one below that is the best fit for your research. If you are not sure, read the appropriate sections before making your selection.

☒ Life sciences ☐ Behavioural & social sciences ☐ Ecological, evolutionary & environmental sciences

For a reference copy of the document with all sections, see nature.com/documents/nr-reporting-summary-flat.pdf

Life sciences study design

All studies must disclose on these points even when the disclosure is negative.

Sample size	No statistical methods were used to predetermine sample size. For in vitro experiments, a minimum of three independent experiments were performed.
Data exclusions	No data were excluded from the analyses.
Replication	Experiments were performed a minimum of three independent times. For cell viability assays, these were performed on three independent occasions in triplicate. All replicates are included within the manuscript.
Randomization	Samples were blindly randomised for inclusion within experiments.
Blinding	All authors were not blinded during in vitro data collection.

Reporting for specific materials, systems and methods

We require information from authors about some types of materials, experimental systems and methods used in many studies. Here, indicate whether each material, system or method listed is relevant to your study. If you are not sure if a list item applies to your research, read the appropriate section before selecting a response.

Materials & experimental systems

n/a	Involved in the study	
☐	☒	Antibodies
☐	☒	Eukaryotic cell lines
☒	☐	Palaeontology and archaeology
☒	☐	Animals and other organisms
☒	☐	Human research participants
☒	☐	Clinical data
☒	☐	Dual use research of concern

Methods

n/a	Involved in the study	
☒	☐	ChIP-seq
☒	☐	Flow cytometry
☒	☐	MRI-based neuroimaging

Antibodies

Antibodies used	Primary antibodies: anti-COMMD4 (Abcam, ab115169 and Bioss, bs-8037R), anti-Histone H3 (Sigma-Aldrich, H0164), anti-nucleolin (Cell Signaling, 12247S), anti-γH2AX (Abcam, ab26350), anti-FLAG (Sigma, F1804), anti-MDC1 (Sigma, PLA0016), anti-p53 Serine 15 (Cell Signaling, 9284), anti-p53 clone D0-7 (Sigma-Aldrich, p8999), anti-Chk2 Threonine 68 (Cell Signaling, 2661), anti-Chk2 (Cell Signaling, 2662), anti-ATM Serine 1981 (Cell Signaling, 13050), anti-ATM (Cell Signaling, 2873), anti-β-actin (BD Biosciences, 612656), anti-H2B (Abcam, ab1790), anti-ubiquityl-histone H2B (Lys120) (Cell Signaling, 5546), anti-RNF20 (Abcam, ab32629 and Merck, MABE948), anti-RNF40 (Abcam, ab126959), anti-H2A (Abcam, 88770), anti-53BP1 (Merck-Millipore, MAB3802), anti-ubiquitin (Cell Signaling, 3936) and anti-hSSB1 20-22. For co-immunoprecipitations and in vitro assays, the following IgG controls were used; normal rabbit IgG (Cell Signaling, 2729), normal mouse IgG (Sigma-Aldrich, N103) and normal sheep IgG (Sigma-Aldrich, 12-515).<br>Secondary antibodies for immunoblotting: IRDye® 680CW Donkey anti-rabbit, IRDye® 800CW Donkey anti-mouse and IRDye® 800CW Donkey anti-goat from LI-COR, Inc.<br>Secondary antibodies for immunofluorescence: Alexa Fluor® 488 donkey anti-mouse (A21202), Alexa Fluor® 488 donkey anti-rabbit (A21206), Alexa Fluor® 594 donkey anti-mouse (A21203) and Alexa Fluor® 594 donkey anti-rabbit (A21207) from (Life Technologies).
Validation	Antibody specificity was validated in-house using siRNA or pharmacological techniques.

Eukaryotic cell lines

Policy information about cell lines

Cell line source(s)	HEK293T, HeLa and U2OS cell lines were purchased from the American Type Culture Collection (ATCC). DRGFP cells were a kind gift from Rodrigue, A. et al. EMBO J 25, 222-231, doi:10.1038/sj.emboj.7600914 (2006).

Authentication	Frozen stocks of all cell lines were made within three passages of purchase from vendors. All experiments were performed on cells grown from original stocks. To verify technique, several cell lines were STR profiled at the Genomics Research Centre (Brisbane, Australia).
Mycoplasma contamination	All cell lines were tested fortnightly and all tested negative for mycoplasma.
Commonly misidentified lines (See ICLAC register)	*Name any commonly misidentified cell lines used in the study and provide a rationale for their use.*
